# A novel penicillin-binding protein inhibitor with unprecedented intracellular activity eradicates multiple pathogenic bacteria

**DOI:** 10.1371/journal.ppat.1014242

**Published:** 2026-07-16

**Authors:** Kai-Xuan Guo, Hong-Xia Hu, Zhen Cheng, Jian-Dong Zhang, Feng-Dong Zhu, Ying-Lei Zhang, Chao-Yue Guo, Kai-Xin Wu, Guo-Hua Cai, Hong-Rong Hong, Xiao-Yu Wan, Xu Yan, Wei Chen, Xuan Liang, Long-Chuan Duan, Huan-Chun Chen, Zheng-Fei Liu

**Affiliations:** 1 National Key Laboratory of Agricultural Microbiology, Huazhong Agricultural University, Wuhan, China; 2 The Cooperative Innovation Center for Sustainable Pig Production, College of Veterinary Medicine, Huazhong Agricultural University, Wuhan, China; 3 Key Laboratory of the Development of Veterinary Diagnostic Products, College of Veterinary Medicine, Huazhong Agricultural University, Wuhan, China; 4 Hongshan Laboratory, Wuhan, China; Universitat fur Weiterbildung Krems, AUSTRIA

## Abstract

The escalating crisis of antibiotics against Gram negative bacteria resistance demands innovative strategies to combat recalcitrant intracellular pathogens. We reposition RS 17053, an α1A-adrenoceptor antagonist with unexplored antibacterial function, as a mechanistically distinct broad-spectrum antibiotic. We proved RS 17053 has strong binding ability with Class A penicillin binding proteins (PBPs), which overcomes the β-lactam barrier by penetrating host cells and disrupts the biosynthesis of peptidoglycan by uniquely targeting MrcA/MecA. RS 17053 eradicated Gram-negative (*Brucella*, *Salmonella*) and Gram-positive (Methicillin resistant *Staphylococcus aureus*, MRSA) pathogens *in vitro* (MICs 0.25–4 μg/mL) and eliminated intracellular *Brucella* within macrophages at 8 μg/mL. Crucially, upon serial passaging under drug pressure, RS 17053 showed a slower progression to high-level resistance. In murine zoonotic infection models, 10 mg/kg/day RS 17053 achieved higher survival rate by caused a 2-log CFU/mL reduction in organs and normalizing pro-inflammatory cytokines. Multi-omics and phenotypic analyses confirmed selective PBP targeting, causing peptidoglycan collapse and bacterial lysis. This work has provided a promising therapeutic against intractable intracellular infections.

## Author summary

The rise of bacteria that are resistant to multiple antibiotics is a major global health crisis. This problem is even worse for infections caused by Gram negative bacteria that can hide and survive inside human cells, where many common drugs cannot reach them effectively. In this study, we discovered that a compound called RS 17053, originally developed for a different purpose, acts as a powerful new type of antibiotic (Striking image). We found that it works by blocking a key bacterial protein involved in building the cell wall, a mechanism different from most current antibiotics. Crucially, RS 17053 can enter mammalian cells to kill stubborn intracellular pathogens like *Brucella* and *Salmonella*. It also shows effectiveness against dangerous drug-resistant bacteria like MRSA. In mouse models of infection, treatment with RS 17053 greatly reduced the number of bacteria in organs and improved survival. Our work introduces RS 17053 as a promising candidate for treating difficult intracellular infections and provides a new strategy for combating antibacterial resistance.

## Introduction

The relentless rise of antibacterial resistance (AMR) poses an existential threat to global health. It is projected to cause 39 million deaths between 2025 and 2050, imposing a disproportionately high burden on low- and middle-income countries, particularly in sub-Saharan Africa and South Asia [1–3]. Zoonotic bacteria like *Brucella* and MRSA evade conventional therapies by persisting intracellularly and acquiring resistance genes [4–6]. While β-lactam antibiotics target PBPs to disrupt peptidoglycan biosynthesis, their inability to penetrate mammalian cells renders them ineffective against intracellular bacteria [7]. Moreover, mutations in Class A PBPs, essential for peptidoglycan transglycosylation and transpeptidation, have spawned untreatable “superbugs” [8–10]. Studies illustrates that Class A PBPs are indispensable virulence determinants for *Brucella*, underpinning its cell wall integrity, intramacrophage survival, and replication within the endoplasmic reticulum-derived niche [4,11].

Drug repurposing offers a promising strategy to circumvent the protracted timeline (exceeding 100 months from Phase 1 to filing) and high costs (averaging $2.23 billion per drug) associated with *de novo* antibiotic development [12–14]. Although high-throughput screening (HTS) of bioactive libraries has identified clinically approved drugs with unexpected, off-target antibacterial effects, yet none have successfully targeted Class A PBPs to eradicate intracellular pathogens [15,16]. The structural plasticity of Class A PBPs (e.g., MrcA in Gram-negatives, MecA in MRSA) provides unexploited binding sites distinct from β-lactam interaction regions [17,18]. However, identifying compounds that concurrently penetrate host cells, resist enzymatic degradation, and avoid efflux pumps remains a formidable challenge [19].

Here we reposition RS 17053 hydrochloride (RS 17053), a selective α1A-adrenoceptor antagonist [20] with no prior antibiotics against Gram negative bacteria applications, as a mechanistically novel Class A PBP inhibitor. A concurrent study reported its ability to disrupt MRSA persisters [21]. However, its molecular target against actively growing bacteria, its precise mechanism of action, and its potential to combat intracellular infections were completely unknown. By integrating in silico docking, multi-omics profiling, and phenotypic validation across zoonotic infection models, we demonstrate its unique capacity to eliminate intracellular pathogens by collapsing peptidoglycan biosynthesis while evading resistance mechanisms that cripple conventional β-lactams.

## Materials and methods

### Ethics statement

All animals were handled strictly with good animal practices according to the Animal Ethics Procedures and Guidelines of the People’s Republic of China. The study was approved by the Animal Ethics Committee of the Huazhong Agricultural University (approval number: HZAUMO-2024–0349 and HZAUMO-2024–0351). All efforts were made to ensure the ethical treatment of all experimental animals in this study and to minimize their suffering.

### Reagents, antibodies, cells and bacteria

Tryptic Soy Agar, Tryptic Soy Broth (236950, 211825 respectively, BD), Luria-Bertani, Müller-Hinton broth (HB0129, HB6231 respectively, HB Biotech), Horse serum (BL209A, Biosharp), Dulbecco’s Modified Eagle Medium (12800–017, Invitrogen), RPMI-1640 (SH30809.01B, HyClone), Gibco FBS (10099–141, Invitrogen), IL-1, IL-6, TNF-α ELISA detection kit (YJ696735, YJ063159, YJ002095 respectively, Yuanji Biotech), Phanta Max Super-Fidelity DNA polymerase (P505-d1, Vazyme), Bacterial Genomic DNA Extraction Kit (DP302–02, Tiangen Biotech), TRIzol (15596018, Thermo Fisher), Agar powder (111860, Biowest), RS 17053 hydrochloride, RFP, Ampicillin, Ceftiofur, Cephalexin, Gentamicin sulfate, Methicillin sodium salt, Propidium Iodide, HADA hydrochloride (HY-101336, HY-B0272, HY-B0522, HY-N7102, HY-B0200, HY-A0276, HY-B0974, HY-D0815, HY-131045 respectively, MCE) were purchased from the specified manufacturers.

Monoclonal antibodies against MecA (orb1821255, Biorbyt), Mouse monoclonal antibodies against IgG (A9044, Sigma), Rabbit monoclonal antibodies against IgG (A0545, Sigma), Mouse monoclonal antibodies against Flag (AP0530, Sigma), Mouse monoclonal antibodies against LPS were prepared in our laboratory.

Mouse macrophage RAW264.7, Human monocyte THP-1 (GDC0143, GDC0100 respectively, China Type Culture Collection).

*B. melitensis* 16M was gifted by Professor Ze-Liang Chen (Shenyang Agricultural University), *B. melitensis* M5-90 was gifted by Professor Chuang-Fu Chen (Shihezi University), *B. melitensis* TZ was isolated from clinical serum samples (Wuwei County, Gansu Province, China) [22], *B. abortus* A19, *B. suis* S2 (from Aolong Biological), *E. coli* ATCC 25922, MRSA ATCC 43300, *S. typhimurium* SL1344 were all gifted by Associate Professor Meng-Hong Dai (Huazhong Agricultural University), *H. parasuis* ATCC70025, *H. parasuis* Nagasaki were both gifted by Associate Professor Xu-Wang Cai (Huazhong Agricultural University), *R. anatipestifer* YM was gifted by Associate Professor Zu-Tao Zhou (Huazhong Agricultural University).

### Plasmids

The MrcA and MecA protein expression clones were synthesized by GenScript. The pET-28a(+) expression plasmids for HIS-tagged MrcA and MecA were constructed using standard molecular biology techniques.

### Molecular docking studies

The software used for high-throughput virtual screening is Schr ödinger Maestro 11.4, and the 3D mapping software is PyMol. *Brucella* MrcA protein was subjected to homology modeling on the SWISS-MODEL website, with a modeling template PDB number of P02918 and a homology of greater than 90%. The FDA approved and biologically active drug library (HY-L001P) used for screening was obtained from MCE (MedChemExpress). Using the Virtual Screening Workflow module for virtual screening, the prepared compounds are imported and molecular docking is performed using the Glide module. Select the optimal compound and conformation based on docking scores and structural principles, and use PyMOL for 3D image drawing.

### Molecular dynamics simulations

After the pre-simulation of the complex, Gromacs2018 was used as the kinetic simulation software, and Amber-ildn was selected as the protein and small molecule force field. TIP3P water was added to the system using the TIP3P model, and a water box with a size of 10*10*10 nm3 (the edge of the water box was at least 1.2 nm away from the protein edge), and an automatic ion equilibrium system was added. Particle-mesh Ewald (PME) deals with electrostatic interactions, using the steepest descent method for energy minimization of the maximum number of steps (50,000 steps). The cut-off distance of the Coulomb force and the van der Waals radius are both 1 nm, and finally the equilibrium system of Regular System (NVT) and Isothermal Isobaric System (NPT) is used, and then the MD simulation of 100 ns is performed at room temperature and pressure. The non-bonding interaction cut-off value is set to 10 Å. The simulated temperature was controlled at 300 K using the V-rescale temperature coupling method and the pressure at 1 bar using the Berendsen method. The built-in analysis module of Gromacs 2018 was used to analyze the simulated trajectories.

### Isothermal titration calorimetry

ITC experiments were conducted using MicroCal ITC at 37 °C, while stirring at 250 rpm in PBS. A control experiment of titrant into buffer was performed to account for the heat of dilution. All titrations were repeated at least three times. For ligand-protein titrations, an approximate protein concentration of 10 μM was used. The concentration of the ligand is approximately ten times higher than that of the protein. All ITC experiments were carried out and analyzed using Launch NanoAnalyze Software.

### Preparation of durg solution

The solvent preparation method is 10% DMSO plus 90% (20% SBE-β-CD in physiological saline). All drugs or compounds are prepared into a solution with a concentration of 4.5 mg/mL and stored in a -80 °C freezer for future use.

### Determination of maximum bacteriostatic diameter

The disk diffusion assay was performed according to the Clinical and Laboratory Standards Institute guidelines (CLSI M45) to determine the inhibitory zones of RS 17053.Briefly, the surface of Mueller-Hinton agar plates was inoculated uniformly with a bacterial suspension adjusted to a 0.5 McFarland standard. Sterile blank filter paper discs (6 mm diameter) were placed on the agar surface. A volume of 10 µL of RS 17053 solution (4.5 mg/mL in 10% DMSO) was applied to each disc, delivering a final amount of 45 µg per disc. RFP susceptibility discs (120 µg/disc) were used as reference controls.

Plates were incubated at 35 ± 2 °C under appropriate atmospheric conditions (aerobic for *Salmonella* and MRSA; 5–10% CO₂ for *Brucella*) for 16–20 hours (*Salmonella*, MRSA) or 48–72 hours (*Brucella*). After incubation, the diameter of the complete inhibition zone around each disc, including the disc diameter, was measured to the nearest millimeter using a caliper. Each test was performed in three independent biological replicates. Results were interpreted according to CLSI breakpoints where available; for RS 17053, the zone diameter was reported in millimeters.

### Drug resistance phenotype detection

The minimum inhibitory concentrations (MICs) of RS 17053 and control antibiotics were determined by the broth microdilution method following the Clinical and Laboratory Standards Institute guidelines (CLSI M07-Ed12).

For *Salmonella* and MRSA, standard cation-adjusted Mueller-Hinton broth (CA-MHB) was used. For *Brucella*, Mueller-Hinton broth was supplemented with 5% defibrinated horse blood. Bacterial suspensions were adjusted to a 0.5 McFarland standard and then diluted in broth to achieve a final inoculum of approximately 5 × 10⁵ CFU/mL in each well.

A stock solution of RS 17053 was prepared in dimethyl sulfoxide (DMSO) and subjected to two-fold serial dilutions in broth within 96-well plates. An equal volume of the standardized inoculum was added to each well. Each plate included growth control (inoculum only) and sterility control (broth only) wells.

Plates were incubated at 35 ± 2 °C for 18–20 hours (*Salmonella*, MRSA) or 48–72 hours (*Brucella*). The MIC was defined as the lowest drug concentration that completely inhibited visible growth. Each assay was performed in three independent biological replicates. Quality control was performed concurrently using *E. coli* ATCC 25922 and *S. aureus* ATCC 29213, with results required to fall within CLSI-specified ranges.

### Growth curves of bacteria

Overnight cultures of *B. melitensis* TZ, *S. typhimurium* SL1344, or MRSA ATCC 43300 were prepared in appropriate broth (MHB for *Salmonella* and MRSA; TSB for *Brucella*). The cultures were standardized to a 0.5 McFarland standard and then diluted 1:100 in fresh pre-warmed broth to achieve a starting inoculum of approximately 1 × 10⁶ CFU/mL.

In a sterile 96-well microplate, 100 µL of bacterial suspension was mixed with 100 µL of broth containing RS 17053 at twice the desired final concentration (ranging from 0× to 4 × MIC). Wells containing bacteria with no drug (growth control) and broth only (blank control) were included on each plate. The plate was covered with a breathable sealing membrane and incubated at 37°C with continuous shaking in a microplate reader (Infinite M200, Tecan). The optical density at 600 nm (OD₆₀₀) of each well was automatically measured at 1-hour intervals for a period of 24 hours. Growth curves were plotted as OD₆₀₀ versus time, and the experiment was performed with three independent biological replicates.

### Time-kill curves

Overnight bacterial cultures were adjusted to a 0.5 McFarland standard in sterile saline and then diluted 1:100 in the appropriate pre-warmed broth (cation-adjusted MHB for *S. typhimurium* and MRSA; TSB for *B. melitensis*) to achieve a starting inoculum of approximately 1 × 10⁶ CFU/mL. RS 17053 was added to the suspensions at final concentrations of 1 × , 2 × , and 4 × the predetermined MIC. A drug-free sample served as the growth control.

The cultures were incubated at 37°C with shaking at 180 rpm. Viable bacterial counts were determined at 0, 0.5, 1, 2, 4, and 8 hours post-exposure. At each time point, 100 µL aliquots were removed, subjected to serial ten-fold dilutions in sterile saline, and plated in triplicate on MHA (*Salmonella*, MRSA) or TSA (*Brucella*) plates. Colony-forming units (CFU/mL) were enumerated after incubation at 37°C for 24 hours (48–72 hours for *Brucella*). Bactericidal activity was defined as a ≥ 3-log₁₀ (99.9%) reduction in CFU/mL compared to the initial inoculum. The experiment was conducted with three independent biological replicates.

### Cytotoxicity test

RAW 264.7 murine macrophages and THP-1 human monocytes were cultured in their respective complete media (e.g., DMEM or RPMI-1640 supplemented with 10% fetal bovine serum) at 37°C in a humidified 5% CO₂ incubator. Cells in the logarithmic growth phase were harvested, counted, and seeded into 96-well plates at a density of 1 × 10⁴ cells per well in 100 µL of medium. After allowing the cells to adhere for 24 hours, the medium was replaced with fresh medium containing RS 17053 at a range of final concentrations (e.g., 0–64 µg/mL). The vehicle solvent (e.g., DMSO) concentration was kept constant (e.g., ≤ 0.5%) across all wells, including the untreated cell control and blank control (medium only).

Following a 24-hour incubation period, 10 µL of the CCK-8 reagent was added directly to each well. The plates were incubated for an additional 1–4 hours, and the absorbance at 450 nm (reference wavelength: 650 nm) was measured using a microplate reader (Infinite M200, Tecan). Cell viability was calculated as a percentage relative to the untreated control cells after subtracting the blank value. The half-maximal cytotoxic concentration (CC₅₀) was determined using non-linear regression analysis (e.g., in GraphPad Prism). Each concentration was tested with a minimum of five technical replicates, and the entire experiment was performed in at least three independent biological replicates.

### Quantitative detection of intracellular sterilization

RAW 264.7 cells were seeded in 24-well plates at a density of 5 × 10⁴ cells per well in complete growth medium and incubated overnight to allow adherence. A fresh culture of *B. melitensis* TZ was grown to mid-log phase, washed, and resuspended in antibiotic-free, serum-free medium (e.g., RPMI-1640). The bacterial suspension was added to the cells at a multiplicity of infection (MOI) of approximately 10:1. Plates were centRFPuged at 500 × g for 10 minutes to synchronize infection, followed by incubation at 37°C under 5% CO₂ for 1 hour to facilitate bacterial internalization.

After infection, the supernatant was removed, and the cells were washed gently twice with phosphate-buffered saline (PBS) to remove non-internalized bacteria. To kill any remaining extracellular bacteria, fresh medium containing 100 µg/mL gentamicin was added, and the cells were incubated for 2 hours. Following this antibiotic protection step, cells were washed three times with PBS and replenished with fresh medium containing gentamicin at a lower, non-internalized concentration (e.g., 10 µg/mL) to prevent extracellular growth. RS 17053 was then added at final concentrations ranging from 1 to 16 µg/mL. Infected, untreated cells served as a control.

At designated time points (e.g., 6, 12, 24, 48 hours) post-treatment, the medium was aspirated, and the cells were lysed with 200 µL of sterile 1% Triton X-100 for 5 minutes. The lysates were then serially diluted in PBS, plated in triplicate on Tryptic Soy Agar plates, and incubated at 37°C for 3–4 days to enumerate colony-forming units (CFU). The intracellular bactericidal activity was expressed as the log_10_ reduction in CFU per well compared to the untreated control at each time point. The experiment was performed with three independent biological replicates.

### Indirect immunofluorescence

RAW 264.7 cells grown on glass coverslips in 24-well plates were infected with *B. melitensis* TZ and treated with compounds as described in the intracellular killing assay. After a 24-hour treatment period, cells were fixed with 4% paraformaldehyde in PBS for 15 minutes at room temperature. Following permeabilization with 0.1% Triton X-100 for 10 minutes and blocking with 5% bovine serum albumin (BSA) for 1 hour, cells were incubated overnight at 4°C with a primary antibody against *Brucella* LPS diluted in blocking buffer. After three washes with PBS, cells were incubated for 1 hour at room temperature with an Alexa Fluor 488-conjugated goat anti-mouse IgG secondary antibody diluted 1:500 in PBS. Cell nuclei were counterstained with 1 µg/mL 4’, 6-diamidino-2-phenylindole (DAPI) for 5 minutes. Finally, coverslips were mounted onto glass slides using an anti-fade mounting medium.

Fluorescent images were acquired using an upright fluorescence microscope (e.g., Olympus BX53) equipped with appropriate filter sets for DAPI and FITC/Alexa Fluor 488. Image capture and processing were performed using consistent exposure times and settings across all experimental groups. The fluorescence intensity of positive cells was measured using Fiji ImageJ software.

### Animal studies

A single-dose acute toxicity study was conducted to evaluate the safety profile of RS 17053. SPF female BALB/c mice (6–7 weeks old, approximately 25 g), purchased from the Hubei Provincial Laboratory Animal Center, were housed under standard conditions (temperature: 25 ± 2°C; humidity: 50 ± 10%; 12-hour light/dark cycle) with free access to food and water. Mice were randomly assigned to seven groups (n = 6 per group). Six groups received a single intramuscular injection of RS 17053 at doses of 7, 14, 28, 56, 112, and 224 mg/kg, respectively. The control group received an equivalent volume of the vehicle solvent (e.g., 10% DMSO + 90% [20% SBE-β-CD in saline]).

To evaluate the effect of RS 17053 on blood pressure in mice at drug doses, ten female BALB/c mice were randomly assigned to two groups (n = 5 per group). Treatment group received intramuscular (i.m.) injection of RS 17053 (10 mg/kg/day) for five consecutive days. Control group received an equivalent volume of PBS vehicle via i.m. injection for five days. On Day 5, at 1 hour post the final administration, non-invasive systolic, diastolic, and mean arterial pressures (SBP, DBP, MAP) were measured for each conscious mouse using a validated tail-cuff system (e.g., CODA System, Kent Scientific). For each mouse, three stable and consistent readings were recorded and averaged. The group mean ± SD was then calculated.

Animals were monitored daily for mortality, clinical signs of toxicity (including changes in activity, posture, and fur condition), and body weight for seven days. No mortality or severe adverse effects were observed at any dose tested. On day 7, all mice were euthanized via cervical dislocation under deep anesthesia. Blood was collected for serum biochemistry analysis, and major organs (liver, spleen, kidneys) were harvested, weighed, and fixed in 4% paraformaldehyde for subsequent histopathological examination.

All efficacy studies wereconducted in accordance with relevant guidelines. Housing conditions were as described above. Studies involving *Brucella* melitensis 16M were performed in an Animal Biosafety Level 3 (ABSL-3) facility. SPF female BALB/c mice (6–8 weeks old, 18 ± 2 g) were randomly assigned into four groups (n = 6 per group): (1) Control (uninfected, untreated); (2) Infection control (infected, untreated); (3) RS 17053 treatment (infected, drug-treated); (4)Positive control (infected, treated with a reference antibiotic, e.g., RFPampin at 10 mg/kg/day). Mice were infected as follows: *S. typhimurium* SL1344 (1 × 10⁷ CFU in 100 µL) by oral gavage; MRSA ATCC 43300 (1 × 10⁷ CFU in 100 µL) by intraperitoneal injection; *B. melitensis* 16M (1 × 10⁵ CFU in 100 µL) by intraperitoneal injection. Three hours post-infection, treatment was initiated. Mice in the treatment group received RS 17053 at 10 mg/kg/day via intramuscular injection for five consecutive days. Control groups received equivalent volumes of the vehicle or reference drug.

Mice were monitored daily for survival, clinical scores, and body weight. At 15 days post-infection (or upon reaching predefined humane endpoints), surviving mice were euthanized. Blood was collected for serum cytokine quantification. The liver and spleen were aseptically removed, weighed, and homogenized in sterile PBS. Serial dilutions of homogenates were plated on appropriate agar media for bacterial load determination (CFU per gram of tissue). Portions of tissues were fixed for histopathological analysis (hematoxylin and eosin staining).

### Histopathology

For histopathological evaluation, tissues (liver and spleen) were harvested immediately after euthanasia and fixed in 4% paraformaldehyde in phosphate buffer (pH 7.4) at 4°C for 48 hours. Fixed tissues were then processed through a standard dehydration series in graded ethanol (70%, 80%, 95%, and 100%), cleared in xylene, and embedded in paraffin wax. Serial sections of 5 µm thickness were cut using a rotary microtome (e.g., Leica RM2235). Sections were mounted on glass slides, deparaffinized in xylene, and rehydrated through a descending ethanol series to water. The sections were stained with hematoxylin (e.g., Harris’s hematoxylin) for 5–8 minutes, differentiated in 1% acid alcohol, and blued in Scott’s tap water substitute. Counterstaining was performed with eosin Y solution for 1–3 minutes. After dehydration and clearing, slides were coverslipped with a permanent mounting medium (e.g., Permount). Stained sections were examined under a light microscope (e.g., Olympus BX53) by a pathologist blinded to the experimental groups. Images were captured using a digital camera attached to the microscope (e.g., Olympus DP27). Histopathological lesions, such as inflammatory cell infiltration, necrosis, and granuloma formation, were evaluated and semi-quantitatively scored based on the extent and severity of the changes.

### Multi-omics

To elucidate the transcriptional response to RS 17053, *B. melitensis* TZ and MRSA ATCC 43300 were treated with RS 17053 at 4 × MIC for 3 hours (*B. melitensis* for 12 hours) alongside mock-treated controls. Bacterial cells were harvested by centRFPugation at 8,000 × g for 10 min at 4°C, washed three times with ice-cold PBS (0.01 M, pH 7.4), and snap-frozen. Total RNA was extracted using TRIzol reagent (Invitrogen) according to the manufacturer’s instructions. RNA concentration and purity were assessed using a NanoDrop 2000 spectrophotometer (Thermo Fisher Scientific). RNA integrity was veRFPied by agarose gel electrophoresis and quantified using an Agilent 2100 Bioanalyzer (RIN > 7.0 for all samples). Library construction and paired-end sequencing (2 × 150 bp) were performed on an Illumina NovaSeq 6000 platform by Shanghai Majorbio Bio-Pharm Technology Co., Ltd. Raw reads were quality-filtered using Fastp (v0.23.2) and aligned to the reference genomes (*B. melitensis* 16M, ASM168050v1; *S. aureus* ATCC 43300, ASM25374v1) using HISAT2 (v2.2.1). Differential gene expression analysis was performed with DESeq2 (v1.38.3), considering genes with an adjusted p-value < 0.05 and |log₂(fold change)| > 1 as differentially expressed. KEGG pathway enrichment analysis was conducted using the clusterProfiler R package (v4.6.2).

For proteomic profiling, bacterial pellets from treated and control groups, prepared identically to transcriptomic samples, were lysed in buffer (8 M urea, 1% SDS) by sonication on ice. Protein concentration was determined using a BCA Protein Assay Kit (Pierce). Proteins (100 µg per sample) were reduced with 10 mM TCEP (37°C, 60 min), alkylated with 40 mM iodoacetamide (room temperature, 40 min in the dark), and precipitated with pre-cooled acetone. The protein pellet was digested with sequencing-grade trypsin (Promega) at a 1:50 (w/w) enzyme-to-protein ratio in 100 mM TEAB buffer at 37°C overnight. The resulting peptides were desalted and analyzed by nanoflow liquid chromatography-tandem mass spectrometry (nLC-MS/MS) on an EASY-nLC 1200 system coupled to a Q Exactive HF-X mass spectrometer (Thermo Fisher Scientific). Peptides were separated on a C18 column with a 120-min gradient of 4–80% acetonitrile in 0.1% formic acid. MS raw data were processed using MaxQuant (v2.1.3.0) against the respective species-specific UniProt databases. Protein identification required at least one unique peptide. Label-free quantification was performed, and proteins with a fold change >1.5 or <0.67 and a p-value < 0.05 (Student’s t-test) were considered significantly altered. Functional enrichment analysis was performed using the KEGG database.

### RT-qPCR and Western blot analysis

RT-qPCR: *B. melitensis* TZ and MRSA ATCC 43300 were grown to mid-log phase and treated with RS 17053 at 4 × MIC for 12 h (*Brucella*) or 3 h (MRSA) at 37°C. Total RNA was extracted using TRIzol reagent (Invitrogen), treated with DNase I (Thermo Fisher), and reverse-transcribed using HiScript III RT SuperMix (Vazyme). Real-time PCR was performed on a LightCycler 480 system (Roche) using SYBR Green Master Mix (Vazyme). The following genes were analyzed: for *B. melitensis*, *mrcA* (primary target), *omp19* (outer membrane protein, used as a stable reference gene based on RNA-seq showing no significant change), and *oppF* (involved in energy metabolism); for MRSA, *mecA* (primary target), *blaZ* (β-lactamase, downregulated in proteomics), and *EF-Tu* (*tuf*) (negative control). Primer sequences are listed in S4 Table. For *Brucella*, relative expression was normalized to *omp19*. For MRSA, normalization was performed using *EF-Tu*, whose Ct values varied by less than 0.5 cycles across all conditions. The 2 ⁻ ΔΔCt method was used to calculate relative expression. Each condition was tested in three independent biological replicates with technical triplicates. Statistical significance was determined by unpaired two-tailed Student’s t-test.

Western blot: MRSA ATCC 43300 was treated with RS 17053 at 4 × MIC for 3 h at 37°C. Bacterial pellets were resuspended in RIPA buffer (50 mM Tris-HCl pH 7.4, 150 mM NaCl, 1% NP-40, 0.5% sodium deoxycholate, 0.1% SDS) supplemented with protease inhibitor cocktail (Roche) and lysed by sonication on ice. Lysates were centrifuged (12,000 × g, 10 min, 4°C), and protein concentration was determined by BCA assay (Thermo Fisher). Equal amounts of protein (20 μg per lane) were separated by 10% SDS-PAGE and transferred to PVDF membranes (Millipore). Membranes were blocked with 5% non-fat milk in TBST (20 mM Tris-HCl pH 7.6, 150 mM NaCl, 0.1% Tween 20) for 1 h at room temperature, then incubated overnight at 4°C with primary antibodies: mouse anti-MecA (1:1,000, Abnova, MAB1705) and rabbit anti-EF-Tu (1:5,000, Abclonal, A17857) as a loading control. After washing, membranes were incubated with HRP-conjugated secondary antibodies: goat anti-mouse IgG (1:5,000, Sigma, A9044) and goat anti-rabbit IgG (1:5,000, Sigma, A0545). Bands were visualized using ECL substrate (Thermo Fisher) and imaged with a ChemiDoc MP system (Bio-Rad). Densitometry was performed using ImageJ (NIH). Three independent biological replicates were analyzed, and representative blots are shown.

### Membrane integrity assay

Mid-log phase cultures of *B. melitensis* TZ, *S. typhimurium* SL1344, or MRSA ATCC 43300 were harvested, washed, and resuspended in phosphate-buffered saline (PBS, pH 7.4) to a standardized density (e.g., approximately 1 × 10⁸ CFU/mL). Bacterial suspensions were treated with RS 17053 at a final concentration of 4 × the predetermined MIC, while control suspensions received an equivalent volume of the drug solvent. After incubation in the dark at 37°C for 2 hours with gentle shaking, propidium iodide (Thermo Scientific, P1304MP) was added to each sample at a final working concentration of 10 µg/mL. The samples were further incubated in the dark at room temperature for 20 minutes.

Fluorescence was measured using a microplate reader (Infinite M200, Tecan) with excitation and emission wavelengths set at 535 nm and 615 nm, respectively. Each assay included the following controls for background subtraction and normalization: untreated bacteria (autofluorescence control), bacteria with PI but no drug (baseline membrane integrity), and bacteria lysed with 70% isopropanol (positive control for maximum PI uptake). The relative fluorescence intensity of the treated samples was calculated and expressed as a percentage of the maximum fluorescence signal from the lysed control. Experiments were performed with at least three independent biological replicates.

### Transmission electron microscopy

Bacterial cultures treated with RS 17053 at the MIC for specified durations were collected by centRFPugation and primarily fixed with 2.5% glutaraldehyde in 0.1 M phosphate buffer (pH 7.4) at 4°C for a minimum of 4 hours. Following buffer washes, samples were post-fixed with 1% osmium tetroxide, dehydrated through a graded ethanol series, and embedded in epoxy resin. Ultra-thin sections (approximately 60–80 nm) were prepared using an ultramicrotome (Leica EM UC7). The sections were stained with uranyl acetate and lead citrate and then examined under a transmission electron microscope (Hitachi HT7700) operated at an acceleration voltage of 80 kV. Images were captured in high-contrast (HC) mode. All sample preparation, sectioning, and imaging were conducted by Wuhan Sevier Biotechnology Co., Ltd., following the standard protocols provided.

### HADA fluorescent dye labeled peptidoglycan

Overnight cultures of *S. typhimurium* SL1344 or MRSA ATCC 43300 were diluted in fresh, pre-warmed Mueller-Hinton Broth to an OD₆₀₀ of 0.1 and grown to mid-log phase (OD₆₀₀ ~ 0.4). Bacteria were then treated with RS 17053 at the MIC or sub-MIC concentrations for 1–2 hours. Following treatment, 500 µL aliquots of the bacterial cultures were transferred to microcentRFPuge tubes. HADA, from a 50 mM stock solution in DMSO, was added to a final concentration of 250 µM. The samples were incubated in the dark at 37°C with shaking for 30 minutes. The labeling reaction was stopped by placing the tubes on ice. Bacteria were pelleted by centRFPugation at 13,000 × g for 2 minutes at 4°C and washed twice with 1.5 mL of ice-cold phosphate-buffered saline (PBS, pH 7.4) to remove excess fluorescent probe. The final bacterial pellet was resuspended in a fixation solution containing an equal volume of PBS and 3% paraformaldehyde (final concentration 1.5%) and incubated for 15 minutes at room temperature in the dark. Fixed cells were washed once with PBS and finally resuspended in a small volume of PBS. A 3 µL aliquot of the suspension was applied to a microscope slide coated with a thin layer of 1% agarose and covered with a coverslip. Imaging was performed using a laser scanning confocal microscope (Olympus FV3000) equipped with a 405 nm laser for HADA excitation (emission collected at 460 nm) and differential interference contrast (DIC) optics. Images were captured and processed using the microscope’s accompanying software (FV31S-SW). For each condition, images from multiple fields of view were analyzed to assess the fluorescence intensity and localization patterns of HADA incorporation.

### Measurement of intracellular ATP levels

Mid-log phase cultures of *S. typhimurium* SL1344 and MRSA ATCC 43300 were adjusted to 1 × 10⁸ CFU/mL in fresh Mueller-Hinton broth and treated with RS 17053 at 2 × MIC or an equivalent volume of vehicle (control) at 37°C with shaking for 2 hours. Bacteria were harvested by centrifugation (8,000 × g, 5 min, 4°C), washed twice with ice-cold PBS, and resuspended in lysis buffer provided with the BacTiter-Glo Microbial Cell Viability Assay kit (Promega, G8230). Luminescence was measured using a microplate reader (Infinite M200, Tecan) after a 5-minute incubation at room temperature. ATP concentrations were calculated from a standard curve using serial dilutions of ATP standard, and values were normalized to total protein content determined by BCA assay (Thermo Fisher). Each condition was tested in three independent biological replicates.

### Measurement of reactive oxygen species (ROS)

Bacterial cultures were prepared and treated as described above. After treatment, cells were harvested, washed twice with PBS, and adjusted to 1 × 10⁷ CFU/mL. Then, 10 μM 2′,7′-dichlorodihydrofluorescein diacetate (DCFH-DA; Sigma-Aldrich, D6883) was added, and the mixtures were incubated at 37°C for 30 min in the dark. Fluorescence was measured at excitation/emission wavelengths of 485 nm and 535 nm, respectively, using a microplate reader. Net fluorescence was calculated by subtracting blank values (PBS containing DCFH-DA without bacteria). ROS levels were expressed as fold change relative to vehicle-treated controls. H₂O₂ (50 μM) treatment for 30 min served as a positive control. Each condition was tested in three independent biological replicates.

### Resistance induction assay

For each strain, a culture was initiated from a single colony and grown to the log-phase in Mueller-Hinton broth (MHB) at 37°C with shaking at 180 rpm. This culture was then used to inoculate (1:1000 dilution) fresh MHB containing RS 17053 at a concentration equivalent to one-fourth of its predetermined minimum inhibitory concentration. This culture was incubated under the same conditions for 24 hours. Daily, the process was repeated for 20 consecutive days by diluting the previous day’s culture into fresh, drug-containing broth, thereby maintaining continuous sub-MIC pressure. The MIC of RS 17053 against the passaged populations was determined using the standard broth microdilution method (CLSI M07) every passage. The fold-change in MIC relative to the baseline MIC of the parental strain (passage 0) was calculated and plotted against the passage number. The experiment was conducted with two independent biological lineages for each bacterial strain.

### Amino acid point mutation and binding affinity validation

Based on molecular docking and sequence conservation analysis, point mutations were introduced to substitute Gln-398 with Arg in *Brucella* MrcA (MrcA-Q398R) and Glu-380 with Cys in MRSA MecA (MecA-E380C). The mutant alleles were synthesized *in vitro* (e.g., by GenScript) and cloned into a pET-28a(+) expression vector, incorporating an N-terminal hexahistidine (His₆) tag for puRFPication. The recombinant plasmids were transformed into *E. coli* BL21(DE3) competent cells for protein expression. A single transformed colony was inoculated into Luria-Bertani (LB) medium containing 50 µg/mL kanamycin and grown at 37°C until the optical density at 600 nm (OD₆₀₀) reached 0.6-0.8. Protein expression was induced by adding isopropyl β-d-1-thiogalactopyranoside (IPTG) to a final concentration of 0.5 mM, followed by further incubation at 16°C for 16–18 hours. Cells were harvested by centRFPugation, resuspended in lysis buffer (e.g., 50 mM Tris-HCl, 300 mM NaCl, 10 mM imidazole, pH 8.0), and lysed by sonication on ice. The His₆-tagged recombinant proteins were puRFPied from the claRFPied lysate using nickel-nitrilotriacetic acid (Ni-NTA) affinity chromatography. After washing with lysis buffer containing 50 mM imidazole, the bound proteins were eluted with elution buffer containing 250 mM imidazole. The eluted proteins were subsequently desalted into a storage buffer (e.g., 50 mM Tris-HCl, 150 mM NaCl, pH 8.0) using a PD-10 desalting column. Protein concentration was determined by the Bradford assay, and purity was confirmed to be > 95% by SDS-polyacrylamide gel electrophoresis (SDS-PAGE) followed by Coomassie Brilliant Blue staining.

### Construction of *mrcA* deletion mutant in *B. suis* S2

An *mrcA* deletion mutant was constructed using homologous recombination. Briefly, upstream and downstream flanking regions (~800 bp each) of mrcA were amplified from *B. suis* S2 genomic DNA and cloned into the pSK2 suicide vector flanking a kanamycin resistance cassette. The construct was electroporated into *B. suis* S2, and double-crossover mutants were selected on TSA plates containing kanamycin (50 μg/mL) and 5% sucrose. Deletion of *mrcA* was confirmed by PCR and sequencing. MIC of RS 17053 against the *ΔmrcA* mutant was determined by broth microdilution as described above. All experiments were performed in three independent biological replicates.

### Assessment of MIC under modified conditions

To evaluate the stability of RS 17053 activity, We conducted MIC testing under different conditions again, referring to the method outlined in the “Drug resistance phenotype detection” section above. First, to assess the inoculum effect, the initial bacterial inoculum was increased to approximately 5 × 10⁷ CFU/mL (100-fold higher than the standard 5 × 10⁵ CFU/mL). Second, to evaluate serum interference, the culture medium was supplemented with 10% fetal bovine serum (FBS). Third, to assess drug stability, RS 17053 stock solutions were diluted in culture medium and pre-incubated at either 4°C or 37°C for 7 days prior to MIC determination against fresh bacterial inocula. All other procedures followed the standard broth microdilution method as described above. Each condition was tested in triplicate biological replicates.

### Statistical analyses

All statistical analyses were performed using GraphPad Prism (version 9.4.1). Data are presented as mean ± SD unless otherwise noted. The normality of data distribution was assessed using the Shapiro-Wilk test. For comparisons between two groups, unpaired two-tailed Student’s t-test (for parametric data) or Mann-Whitney U test (for non-parametric data) was used. For comparisons among more than two groups, one-way or two-way analysis of variance (ANOVA) was performed, followed by Dunnett’s or Tukey’s post hoc test for multiple comparisons, as appropriate. P-values are reported numerically in figures, with significance thresholds indicated in legends. No data were excluded from analyses except in cases of technical failure, which were documented.

For mouse infection models, group sizes (n = 5–6) were determined based on: (a) our extensive prior experience with the *Brucella*, *Salmonella*, and MRSA models, where this sample size reliably detects significant differences in bacterial burden (≥1 log10 CFU/organ) with 80% power and alpha = 0.05 [23,24]; and (b) ethical principles of the 3Rs (Reduction) to use the minimum number of animals necessary to achieve scientific objectives [25]. No formal a priori power calculation was performed for exploratory endpoints like cytokine levels, which were analyzed as secondary outcomes.

## Results

### Identification and validation of RS 17053 as a novel potent Class A PBPs inhibitor

**Fig 1:**
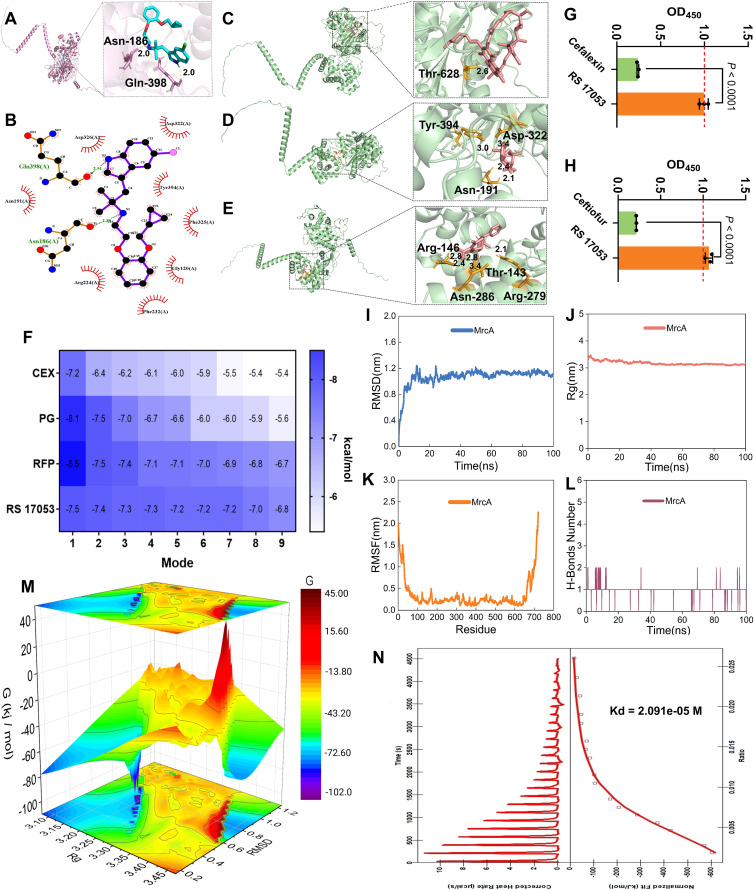
Identification and validation of a novel Class A penicillin-binding protein-targeting compound for a Achilles’ Heel of *Brucella.* **(A)** Simulated image of the RS 17053 at 4 Å drug binding domain (DBD) in MrcA (PDB: 3D0F) generated by PyMOL software, with residues Asn-186 and Gln-398 shown in pink. **(B)** Two-dimensional plan view of RS 17053 with 4 Å DBD in MrcA generated by PyMOL software. **(C)** Simulated image of the RFP at 4 Å DBD in MrcA generated by PyMOL software, with residues Thr-628 shown in yellow. **(D)** Simulated image of the PG at 4 Å DBD in MrcA generated by PyMOL software, with residues Asn-191, Asp-322 and Tyr-394 shown in yellow. **(E)** Simulated image of the CEX at 4 Å DBD in MrcA generated by PyMOL software, with residues Thr-143, Arg-146, Arg-279 and Asn-286 shown in yellow. **(F)** Thermograms of binding energies for the top 9 best binding models of different drugs and MrcA protein. **(J-H)** Enzyme linked immunosorbent assay (ELISA) for detecting the correlation between RS 17053 and Beta-lactam antibiotics (n = 3, biological replicates). **(I-L)** Root mean square deviation (RMSD), Root mean square fluctuation (RMSF), Radius of gyration (Rg) and H-bonds number of RS 17053 and the MrcA complexes in a 100 ns molecular dynamics (MD) simulation. **(M)** Molecular dynamics simulation of the binding free energy morphology of RS 17053 and MrcA at 100ns. **(N)** Determination of Affinity between RS 17053 and MrcA by Isothermal Titration Calorimetry. Data are expressed as mean ± s.d. Significant differences between two groups were determined using unpaired two-tailed Student’s t-test **(P)**. Exact P-values from the indicated statistical tests are shown on the figures, ns, not significant (*P* ≥ 0.05).

We sought to identify novel non-β-lactam compounds targeting the Class A PBP (MrcA) from a library of over 50,000 known biologically active and natural products, an essential enzyme for peptidoglycan biosynthesis and a known Achilles’ heel of *Brucella* (Fig A in S1 File). Through a structure-based high-throughput virtual screening approach, we identified a lead compound among the 150 potential compounds preliminarily screened (S1 Table), RS 17053 (Fig B in S1 File), which demonstrated a high predicted binding activity to the active site of *Brucella* MrcA. Molecular docking revealed that RS 17053 forms stable interactions within the 4 Å drug-binding domain (DBD) of MrcA, involving key residues such as Asn-186 and Gln-398 (Fig 1A and 1B). Comparative simulations with known antibiotics (RFP, RFP; Penicillin G, PG; Cefotaxime, CEX) highlighted the stable and unique binding ability and mode of RS 17053 (Fig 1C-1F). Importantly, ELISA assays confirmed that RS 17053 does not cross-react with β-lactam antibiotics, indicating a unique mechanism of action (Fig 1G and 1H). Compared to Cefalexin, RS 17053 showed a significantly higher signal (mean difference = 0.753 OD, 95% CI [0.671, 0.836]; t(4) = 25.27, p < 0.0001). Compared to Ceftiofur, RS 17053 also showed a large increase (mean difference = 0.854 OD, 95% CI [0.787, 0.922]; t(4) = 35.06, p < 0.0001). The superior binding stability of RS 17053 and MrcA complex was confirmed by molecular dynamics (MD) simulations, which showed low root mean square deviation (RMSD), minimal fluctuation (RMSF), stable radius of gyration (Rg), and consistent hydrogen bond formation over a 100 ns trajectory (Fig 1I and 1M). Isothermal titration calorimetry (ITC) quantified the strong binding affinity between RS 17053 and purified MrcA protein (Figs 1N and C in S1 File).

### RS 17053 exerts potent bactericidal activity against *Brucella in vitro* and eradicates intracellular infection

**Fig 2:**
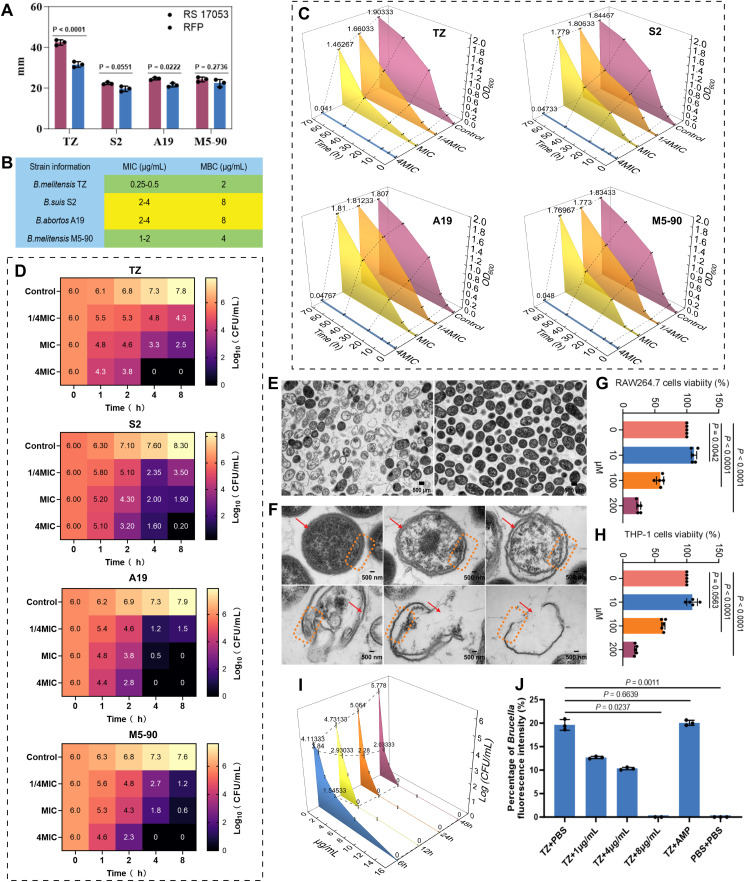
RS 17053 exhibits potent bactericidal activity against various *Brucella* species *in vitro* and can enter cells to eliminate *Brucella.* **(A)** Determination of the maximum inhibitory diameter of RS 17053 against various *Brucella* species by drug sensitive paper diffusion method (n = 3, biological replicates). **(B)** Determination of the minimum inhibitory concentration of RS 17053 against various *Brucella* species by micro broth dilution method (n = 3, biological replicates). **(C)** Growth inhibition ability of RS 17053 with different MIC concentrations on different species of *Brucella* (n = 3, biological replicates). **(D)** Kill-time curves of RS 17053 with different MIC concentrations on different species of *Brucella* (n = 3, biological replicates). **(E)** Observation of the effect of RS 17053 on the morphology and structure of *B. melitensis* TZ at MIC concentration using transmission electron microscopy. Scale bar, 500 μm. **(F)** Observation of the dynamic lysis process of *B. melitensis* TZ by transmission electron microscopy (TEM) under MIC inhibitory concentration of RS 17053. The yellow dashed box and red arrow highlight the compromised cell wall membrane structure. Scale bar, 500 nm. **(G-H)** Toxicity of different concentrations of RS 17053 on RAW264.7 cells and THP-1 cells (n = 5, technical replicates). **(I)** Colony-forming unit measurement of intracellular *B. melitensis* TZ in the presence of RS 17053 in RAW264.7 cells (n = 3, biological replicates). **(J)** Ability of different concentrations compounds or drugs to clear *B. melitensis* TZ in RAW264.7 cells at 24 hours. Data are expressed as mean ± s.d. Statistical significance among groups was assessed by one-way ANOVA (C, D, I, J) followed by Dunnett’s post-hoc test. Significance was determined using unpaired two-tailed Student’s t-test (A, G, H) for comparisons between two specified groups. Exact P-values from the indicated statistical tests are shown on the figures, ns, not significant (*P* ≥ 0.05).

We next evaluated the anti-*Brucella* efficacy of RS 17053 *in vitro*. The compound exhibited significant zones of inhibition and low minimum inhibitory concentrations (MICs) against various *Brucella* species, including clinical isolate *B. melitensis* TZ, vaccine strains M5 (*ovis*), S2 (*suis*), and A19 (*bovis*) (Figs 2A, 2B and D in S1 File and S2 Table). Growth inhibition assay and time-kill assays confirmed its rapid and concentration-dependent bactericidal activity, reducing bacterial counts by >3 log10 CFU/mL within 8 hours (Fig 2C and 2D). TEM of *Brucella* treated with RS 17053 showed severe morphological damage, including cell wall rupture and dissolution (Fig 2E and 2F), a phenotype consistent with that observed for the β-lactam antibiotic AMP (Fig F in S1 File). Critically, RS 17053 effectively eliminated intracellular *B. melitensis* TZ residing within RAW264.7 macrophages, significantly reducing bacterial loads without inducing cytotoxicity at effective concentrations (Figs 2G, 2J and E in S1 File).

### RS 17053 is well tolerated and confers robust protection in a model of *Brucella* acute infection

**Fig 3:**
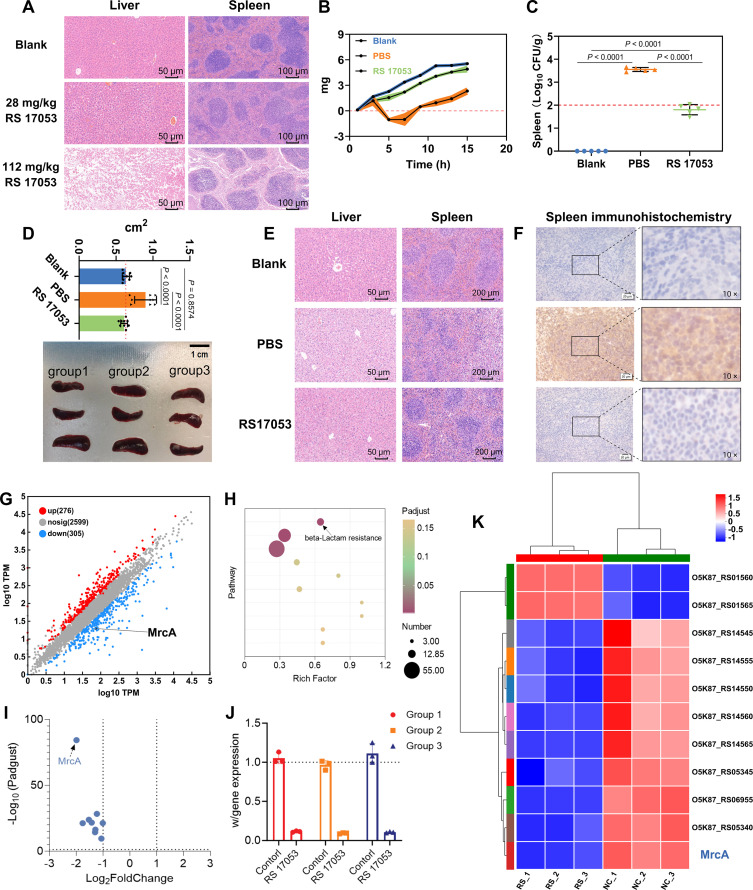
RS 17053 protects mice from *B. melitensis* 16M infection and disrupts bacterial cell wall integrity by inhibiting PBP function. **(A)** Histopathological assessment of the liver and spleen in different groups of mice. Scale bar, liver, 50 μm; spleen, 100μm. **(B)** Record of mouse body weight after a single dose of RS 17053 at 10 mg/kg (n = 5, mice). **(C)** Gross autopsy of spleens of mice infected by *B. melitensis* 16M (n = 3, mice). **(D)** Bacterial load in the spleen of different treatment groups in a mouse model of *B. melitensis* 16M infection (n = 5, mice). **(E)** Histopathological assessment of the livers and spleens in different treatment groups in a mouse model of *B. melitensis* 16M infection. Scale bar of livers, 50 μm; Scale bar of spleens, 200 μm. **(F)** Immunohistochemical evaluation of spleens in different treatment groups in a mouse model of *B. melitensis* 16M infection. Scale bar, 20 μm. **(G)** Volcano plot of differentially-expressed genes (DEGs) with *B. melitensis* TZ transcriptomics. **(H)** *B. melitensis* TZ transcriptome KEGG pathway enrichment bubble map. **(I)** Volcanic map of differential gene expression in beta-lactam resistance pathway in *B. melitensis* TZ transcriptome. **(J)** *B. melitensis* TZ was treated with RS 17053 at 4 × MIC for 12 h**.** Relative expression of mrcA (primary target), OppF (ABC transporter, downregulated in RNA-seq), and Omp19 (stable reference) was determined. Normalization was performed using Omp19 (Ct unchanged). **(K)** Clustering heatmap of some important DEGs. Data are expressed as mean ± s.d. Statistical significance among treatment groups was assessed by one-way ANOVA. (C) followed by Tukey’s post-hoc test for all pairwise comparisons. Statistical significance among groups was assessed by one-way ANOVA. (B) followed by Dunnett’s post-hoc test. Exact P-values from the indicated statistical tests are shown on the figures, ns, not significant (*P* ≥ 0.05).

We first evaluated the acute toxicity of RS 17053 in healthy BALB/c mice. Intramuscular administration of ascending doses revealed a favorable safety profile, with no adverse effects on survival or viscera (Fig G in S1 File) at the therapeutic dose of 28 mg/kg once daily. Furthermore, no significant changes in serum levels of pro-inflammatory cytokines (IL-1β, IL-6, TNF-α) or alkaline phosphatase (AKP) were observed (Fig G in S1 File), and histopathological assessment confirmed the absence of lesions in the liver and spleen (Fig 3A), establishing a wide safety margin for subsequent therapeutic studies. Given the origin of RS 17053 as an α1A-adrenoceptor antagonist, we specifically evaluated its potential to induce hypotension at the therapeutic dose (S2 Table). Mice treated with RS 17053 (10 mg/kg/day, i.m., for 5 days) showed no significant changes in systolic, diastolic, or mean arterial blood pressure compared to vehicle-treated controls (all P > 0.38) [26].

The *in vivo* efficacy of RS 17053 was then assessed in a acute infection model infected with the virulent *B. melitensis* 16M strain (Fig H in S1 File). Treatment with RS 17053 (10 mg/kg) significantly increased the weight of mice compared to the infected untreated group (Fig 3B), ameliorated splenomegaly (Fig 3C), and reduced bacterial burdens in the spleen by >2 log10 CFU/mL (Fig 3D). Statistical analysis revealed a large effect size for the reduction in splenic bacterial load (RS 17053 vs. PBS: Cohen’s d = 2.87 [95% CI: 1.45–4.29]. The treatment also mitigated infection-induced pathological injury, as shown improvement in histopathological lesions and inflammatory infiltration in the livers and spleens (Fig 3E). Immunohistochemistry confirmed a substantial decrease in bacterial load in the spleens of treated mice (Fig 3F), corroborating the compound’s potent *in vivo* bactericidal activity.

### Broad-spectrum antibacterial activity of RS 17053 against Gram-negative and Gram-positive pathogens

**Fig 4:**
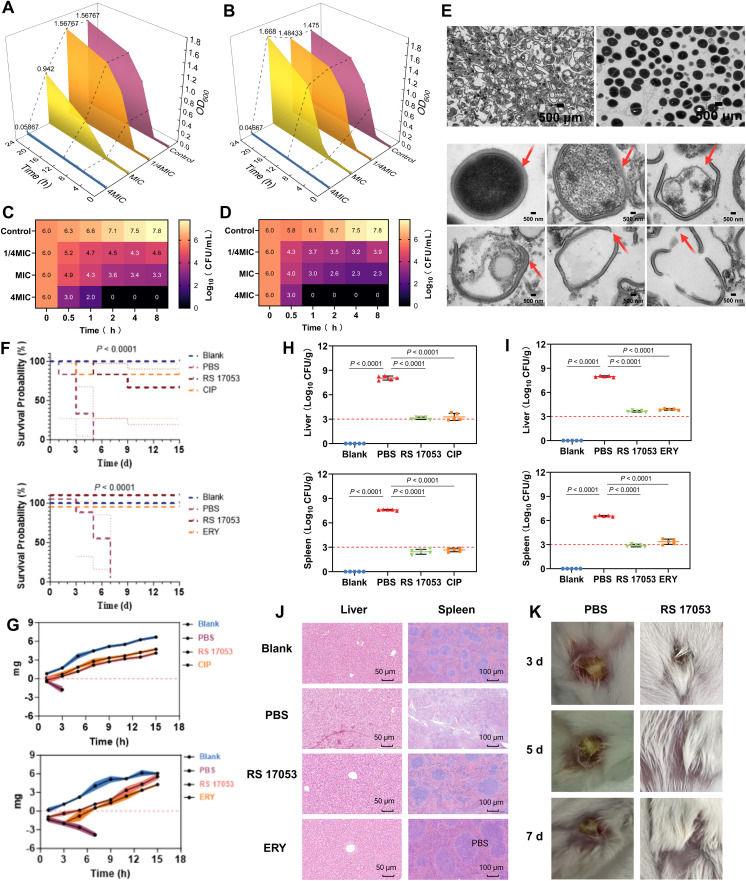
Demonstrating broad-spectrum antibacterial efficacy of RS 17053 against diverse pathogens including MRSA *in vitro* and *in vivo.* **(A-B)** Growth inhibition ability of RS 17053 with different MIC concentrations on *S. typhimurium* SL1344 and MRSA, respectively (n = 3, biological replicates). **(C-D)** Kill-time curves of *S.* *typhimurium* SL1344 and MRSA with different MIC concentrations of RS 17053, respectively (n = 3, biological replicates). **(E)** Observation of the effect of RS 17053 on the morphology and structure of MRSA at MIC concentration, as well as the dynamic lysis process of MRSA under transmission electron microscopy. Red arrow represents the cell wall membrane structure of MRSA. Scale bar, 500 μm and 500 nm. **(F)** Survival curves of mice in an anti-*S. typhimurium and* anti-MRSA infection study, respectively (n = 6, mice). **(G)** Record of mouse body weight after a single dose of RS 17053 at 10 mg/kg (n = 5,mice). (H-I) Bacterial load in the livers and spleens of different treatment groups in a mouse model of *S. typhimurium* SL1344 and MRSA infection (n = 5, mice). **(J)** Histopathological assessment of the livers and spleens in different treatment groups in a mouse model of MRSA infection. Scale bar of livers, 50 μm; Scale bar of spleens, 100 μm. **(K)** Effect of treating MRSA infected mouse skin with RS 17053 for different days. A, C and other groups with CIP are all associated with *S. typhimurium*; B, D and other groups with ERY are all associated with MRSA. Data are expressed as mean ± s.d. Statistical significance among groups was assessed by one-way ANOVA (A, B, C, D) followed by Dunnett’s post-hoc test. Statistical significance among treatment groups was assessed by one-way ANOVA (H, I) followed by Tukey’s post-hoc test. Exact P-values from the indicated statistical tests are shown on the figures, ns, not significant (*P* ≥ 0.05).

We extended our evaluation to other clinically relevant pathogens. RS 17053 exhibited potent growth inhibition and bactericidal effects against Gram-negative *S. typhimurium* SL1344 and Gram-positive MRSA (Fig 4A-4D and S3 Table). TEM confirmed similar cell wall membrane layer damage in MRSA (Fig 4E). In murine models of lethal *S. typhimurium* and MRSA infection, RS 17053 treatment significantly improved survival rates (Fig 4F), saved weight (Fig 4G), reduced bacterial loads in the liver and spleen (Fig 4H and 4I), alleviated organ damage (Figs 4J I and J in S1 File), and modulated systemic inflammation, as evidenced by normalized cytokine levels and improved hematological parameters (Fig K in S1 File). The hazard ratio for death in RS 17053‑treated mice compared to PBS‑treated controls was 0.12 [95% CI: 0.03–0.41] for *S. typhimurium* and 0.09 [95% CI: 0.02–0.38] for MRSA (Log‑rank test with Bonferroni correction, both P < 0.001). For *S. typhimurium*, RS 17053 treatment led to a mean reduction of 4.5 log10 CFU/liver compared to PBS (Cohen’s d = 3.21 [95% CI: 1.89–4.53]; for MRSA, the reduction was 3.8 log10 CFU/spleen (Cohen’s d = 2.94 [95% CI: 1.67–4.21]), both indicating very large effects. In a relatively short period of time, RS 17053 also promoted healing of MRSA-infected skin wounds (Fig 4K).

### Mechanistic insights: RS 17053 inhibits peptidoglycan biosynthesis by targeting Class A PBPs

**Fig 5:**
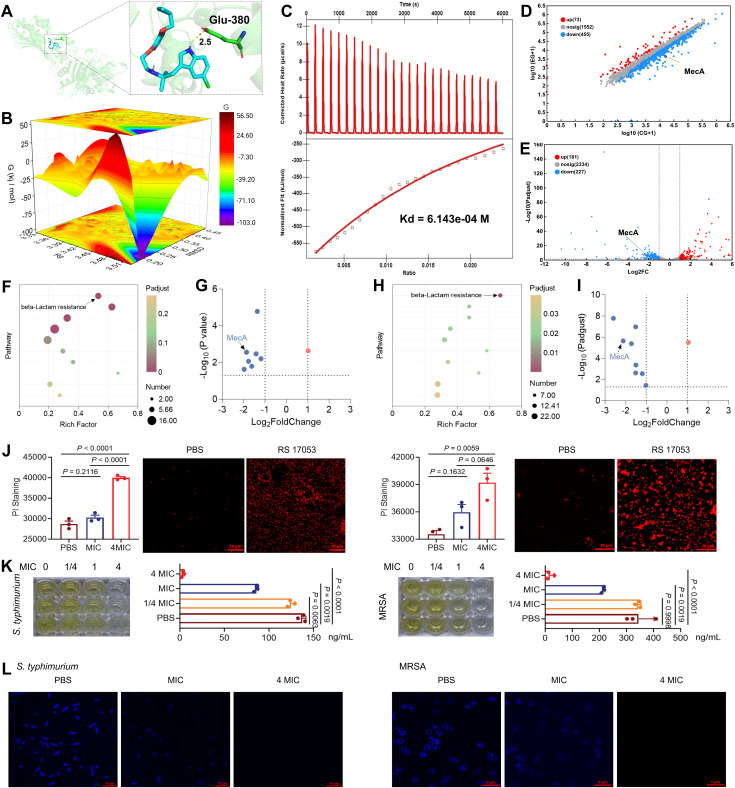
RS 17053 inhibits the expression of bacterial Class A PBPs, thereby hindering the biosynthesis of peptidoglycan, ultimately leading to bacterial lysis and death. **(A)** Simulated image of the RS 17053 at 4 Å DBD in MecA (PDB: 3JTP) generated by PyMOL software, with residues Glu-380 shown in green. **(B)** Molecular dynamics simulation of the binding free energy morphology of RS 17053 and MecA at 100ns. **(C)** Determination of Affinity between RS 17053 and MecA by Isothermal Titration Calorimetry. **(D)** Volcano plot of differentially-expressed proteins (DEPs) with MRSA ATCC43300 proteomics. **(E)** Volcano plot of DEGs with MRSA ATCC43300 transcriptomics. **(F)** MRSA proteome KEGG pathway enrichment bubble map. **(G)** Volcanic map of differential gene expression in beta-lactam resistance pathway in MRSA proteome. **(H)** MRSA transcriptome KEGG pathway enrichment bubble map. **(I)** Volcanic map of differential gene expression in beta-lactam resistance pathway in MRSA transcriptome. **(J)** Damage of RS17053 to *S. typhimurium* and MRSA ATCC43300 were evaluated at different MIC concentration using Live & Dead Bacterial Staining Kit (n = 3, biological replicates). **(K)** Determination of RS 17053 on *S. typhimurium* and MRSA ATCC43300 peptidoglycan damage using bacterial peptidoglycan Elisa assay kit (n = 3, biological replicates). **(L)** *S. typhimurium* SL1344 and MRSA treated with RS 17053 were stained with the fluorescent cell wall marker D-amino acid 7-hydroxycoumarin carbonyl amino-D-alanine (HADA), and analyze by laser confocal microscopy. Scale bar, *S. typhimurium* SL1344, 10 μm; MRSA, 5 μm. Data are expressed as mean ± s.d. Statistical significance among groups was assessed by one-way ANOVA (J, K) followed by Dunnett’s post-hoc test. Exact P-values from the indicated statistical tests are shown on the figures, ns, not significant (*P* ≥ 0.05).

To uncover the potential targets of RS 17053 in anti-*B. melitensis* and anti-MRSA activity, We analyzed the changes in bacterial omics levels after treatment with RS 17053. Transcriptomic analysis of RS 17053-treated *B. melitensis* TZ showed,a total of 581 differentially expressed genes (DEGs) with 276 upregulated and 305 downregulated in the *B. melitensis*, showed significant different expression patterns before and after RS 17053 treatment (FC < 0.5 or >2, P < 0.05). Among these, significant downregulation of peptidoglycan biosynthesis and β-lactam resistance pathways, including the key protein MrcA of Class A PBP in this pathway (Figs 3G-3K and L in S1 File). Molecular simulations and ITC confirmed its high-affinity binding to the Class A PBP (MecA) of MRSA (Figs 5A-5C and M in S1 File), underscoring a conserved mechanism across bacterial species may exist. Therefore, further multi omics analysis of MRSA treated with RS 17053 showed that, in proteomics, compared with simulated treatment bacteria, after RS 17053 treatment, MRSA had a total of 528 DEPs, of which 73 were upregulated and 455 were downregulated, showing a significant expression pattern (FC < 0.5 or>2, P < 0.05); in transcriptomics, there were a total of 181 upregulated and 227 downregulated genes, showing significantly different expression patterns before and after treatment with RS 17053 (FC < 0.5 or>2, P < 0.05). Consistently, omics analysis indicates that profound repression of pathways related to cell wall biosynthesis and β-lactam resistance, including the key protein MecA of Class A PBP in this pathway (Figs 5D-5I, N, O, P and Q in S1 File). Key genes, including MrcA and MecA, were suppressed, as validated by RT-qPCR and Westen blot (Figs 3J, R, and S in S1 File).

This disruption impairs cell wall integrity, evidenced by live/dead staining (Fig 5J), peptidoglycan damage in ELISA assays (Fig 5K), and inhibited nascent peptidoglycan synthesis in HADA staining (Fig 5L). At 4 × MIC, RS 17053 reduced peptidoglycan levels by 98% relative to control in *S. typhimurium* (Cohen’s d = 5.12 [95% CI: 3.01–7.23]) and by 96% in MRSA (Cohen’s d = 4.87 [95% CI: 2.76–6.98]), indicating a very strong effect. In summary, our research has demonstrated that the bactericidal mechanism of RS 17053 is to inhibit the expression of Class A PBPs (MrcA and MecA), essential genes for bacterial peptidoglycan biosynthesis, thereby preventing the formation of a complete wall protective layer and ultimately leading to bacterial lysis and death. The conserved role of Class A PBPs in peptidoglycan synthesis is summarized in Fig T in S1 File.

Analysis of transcriptomic and proteomic data revealed that RS 17053 also downregulates ABC transporters, quorum sensing, while upregulating stress response genes in both *B. melitensis* and MRSA. Phenotypic validation in *S. typhimurium* and MRSA showed that RS 17053 (2 × MIC, 2 h) significantly reduced intracellular ATP levels and increased ROS production (Fig W in S1 File). Collectively, these data demonstrate that RS 17053 not only arrests peptidoglycan synthesis but also triggers a cascade of metabolic collapse and oxidative stress, ultimately contributing to bacterial cell death.

### RS 17053 exhibits a low level of resistance development upon serial passage

**Fig 6:**
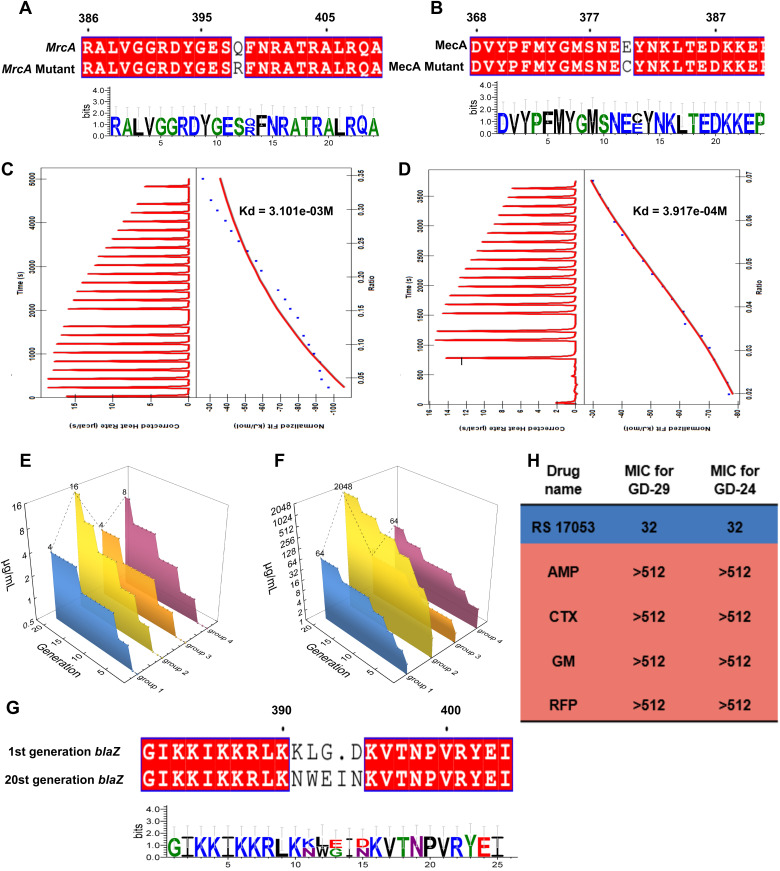
Low resistance propensity of RS 17053 and enhanced druglikeness through AI-guided optimization. **(A)** Amino acid point mutation (Gln mutations to Arg) sequence alignment analysis of MrcA protein and conservatism analysis of MrcA mutation gene sequence. **(B)** Amino acid point mutation (Glu mutations to Cys) sequence alignment analysis of MecA protein and conservatism analysis of MecA mutation gene sequence. **(C)** Isothermal titration calorimetry assay of the affinity of RS 17053 with MrcA after point mutations. **(D)** Isothermal titration calorimetry assay of the affinity of RS 17053 with MecA after point mutations. **(E-F)** MIC values of each generation of strains induced by drug resistance in *B. melitensis* and MRSA were carried out by microbroth dilution method for 20 consecutive generations (n = 3, biological replicates). **(G)** Amino acid mutation sequence alignment analysis of blaZ protein and conservatism analysis of *blaz* gene sequence. **(H)** MIC values of different drugs against clinical isolates of Salmonella GD-29 and GD-24 resistant to beta lactam antibiotics. Data are expressed as mean ± s.d. Statistical significance across passages was assessed by one-way ANOVA (E, F) followed by Dunnett’s post-hoc test. Exact P-values from the indicated statistical tests are shown on the figures, ns, not significant (*P* ≥ 0.05).

The MIC curve variation was assessed by serial passaging of *B. melitensis* and MRSA under sub-MIC RS 17053 pressure for 20 generations. Only a minimal increase in MIC was observed (Fig 6E and 6F), indicating a low resistance risk. After 20 passages, the median MIC increase was 8‑fold for *B. melitensis* (Cohen’s d for log2 MIC = 0.45 [95% CI: –0.12–1.02], P > 0.05) and 32‑fold for MRSA (Cohen’s d = 1.23 [95% CI: 0.56–1.90], P = 0.06), whereas methicillin induced a > 1024‑fold increase. Analysis of the whole genome sequencing results induced by resistance in the 1st and 20th generations showed that only the 291th-294th amino acids of the *blaZ* resistance gene on the MRSA genome plasmid were mutated (Figs 6G and U in S1 File). These results suggest that RS 17053 can delay the development of high-level resistance compared to antibiotics like methicillin, as evidenced by the substantially lower MIC shift after 20 passages. To understand the structural basis, we introduced point mutations (Gln → Arg in MrcA; Glu → Cys in MecA) at conserved binding residues (Figs 6A, 6B and C in S1 File). ITC confirmed that these mutations significantly reduced binding affinity (Fig 6C and 6D), suggesting that resistance-conferring mutations are evolutionarily constrained. To directly test whether MrcA is the functional target of RS 17053 in *Brucella*, we constructed an *mrcA* deletion mutant in *B. suis* S2 by homologous recombination (Fig X in S1 File). The MIC of RS 17053 against the *ΔmrcA* mutant was 8–16 μg/mL, representing a 32- to 64-fold increase compared to the wild-type strain (MIC = 0.25-0.5 μg/mL). To further characterize the robustness of RS 17053’s antibacterial activity under varying physiological and experimental conditions, we assessed the impact of inoculum size, serum protein, and drug pre-incubation temperature on *B. melitensis* TZ and MRSA’s MIC (Fig Y in S1 File). When the initial bacterial inoculum was increased 100-fold, the MIC of RS 17053 against its increased by only 2-fold, indicating a minimal inoculum effect. The presence of 10% fetal bovine serum (FBS) in the culture medium did not alter the MIC for any of the two pathogens, suggesting negligible protein binding interference. Furthermore, pre-incubation of RS 17053 in culture medium at 4°C for 7 days resulted in no change in MIC, whereas pre-incubation at 37°C for 7 days led to a modest 2- to 4-fold increase in MIC, indicating partial temperature-dependent degradation over extended periods. In addition, We evaluated the activity of RS 17053 against a subset of MDR *Salmonella* strains possessing high-level beta-lactam resistance. Two representative clinical isolates from this collection, GD-29 and GD-24, were selected [27]. Both strains exhibit extensive resistance profiles, including high-level resistance to AMP (MIC > 512 µg/mL) as reported in that study. The specific beta-lactam resistance mechanism(s) in these strains were not further characterized in the present study. Notably, RS 17053 demonstrated potent activity against both strains, with a MIC of 32 µg/mL (Fig 6H).

## Discussion

Our study repositions RS 17053, an unexplored α1A-adrenoceptor antagonist, as a bona fide Class A PBP inhibitor that surmounts two fundamental limitations of β-lactam antibiotics: an inability to penetrate mammalian cells and vulnerability to target-site mutations [7,19,28]. By exploiting non-canonical binding pockets in MrcA (ASN-186/GLN-389) and MecA (GLU-380), RS 17053 disrupts peptidoglycan biosynthesis at concentrations 10-fold lower than required for β-lactam-mediated PBP inhibition [29,30]. The bactericidal activity of RS 17053, visualized by TEM, resulted in cell wall disintegration identical to the phenotype caused by the classical β-lactam ampicillin, providing direct cytological evidence for its primary action on peptidoglycan biosynthesis. The collapse of peptidoglycan synthesis further triggered secondary metabolic perturbations, including ATP depletion and oxidative stress, as validated by phenotypic assays in *S. typhimurium* and MRSA. These multifaceted effects likely contribute to the potent bactericidal activity and low resistance propensity of RS 17053. While our multi-omics data also revealed downregulation of PBP3 in MRSA, no other PBPs were significantly altered in *Brucella* beyond MrcA, and molecular docking did not predict binding to Class B PBPs. Thus, the observed changes in other PBPs likely reflect indirect transcriptional responses to cell wall stress rather than direct targeting. It is well established that Class B PBPs (e.g., PBP3) are not typically targeted by β-lactam-like inhibitors and often respond indirectly to cell wall damage through two-component systems [31,32]. We conclude that Class A PBPs are the primary molecular targets of RS 17053. Crucially, RS 17053 achieves intracellular killing concentrations (8 μg/mL in macrophages) that surpass clinical benchmarks for *Brucella* eradication, outperforming aminoglycosides that fail against phagocytosed bacteria [4,28,33]. This discovery addressing a decades-old therapeutic void for zoonotic intracellular reservoirs [33]. At the effective therapeutic dose *in vivo*, RS 17053 showed a safe protective effect, especially with no significant cardiovascular side effects confirmed at the effective dose. This is consistent with the known high subtype selectivity of RS 17053 towards alpha 1A receptors, which are not the main regulators of vascular tone [20,34]. During the preparation of this manuscript, a study by Xu et al. reported that RS 17053 could eradicate MRSA persisters, potentially through membrane-targeting effects [21]. But our work provides a complementary yet distinct mechanistic understanding. Crucially, our discovery of its PBP-targeting mechanism directly explains its potent intracellular activity, which we demonstrated against *Brucella* and *Salmonella*, a therapeutic application not previously envisioned. These findings collectively position RS 17053 as a versatile scaffold with multiple potential antibacterial applications.”

The structural basis of RS 17053’s activity explains its low resistance level. Unlike β-lactams, which select for PBP mutations or β-lactamase expression [10,35,36], RS 17053 maintains activity against mutated PBPs and induces only a modest MIC increase (8–32 fold) after 20 passages. This contrasts sharply with the high-level resistance rapidly developed against methicillin (1024-fold increase), and is comparable to the shift observed for RFP (32-fold). The emergence of a *blaZ* mutation rather than a *mecA* mutation during serial passage in MRSA is noteworthy. RS 17053 is not a β-lactam antibiotic, and BlaZ is a β-lactamase that hydrolyzes the β-lactam ring of penicillins [37]. However, *blaZ* and *mecA* are co-regulated by the BlaR1-BlaI two-component system [38]. The *blaZ* mutation we observed may represent an indirect adaptive response under sub-MIC drug pressure rather than a direct resistance mechanism against RS 17053. Active-site mutations in PBP2a (MecA) that reduce RS 17053 binding would likely compromise its essential transpeptidase activity, imposing a high fitness cost. In contrast, *blaZ* encodes a β-lactamase that degrades certain β-lactams without directly interfering with cell wall synthesis, representing a lower-cost adaptive mechanism under sub-MIC drug pressure [39]. This is consistent with the modest MIC increase (8- to 32-fold) observed after 20 passages. It aligns with emerging evidence that non-β-lactam scaffolds evade classical resistance mechanisms by targeting allosteric PBP sites [8,9,40]. But, the precise mechanism by which this *blaZ* mutation contributes to reduced RS 17053 susceptibility remains to be fully elucidated. Importantly, when we forcibly deleted *mrcA* in *Brucella*, RS 17053 MIC increased by 32- to 64-fold (Fig X in S1 File), directly confirming that Class A PBPs are the genuine functional targets. Our multi-omics analyses further confirm that RS 17053’s bactericidal effects stem specifically from peptidoglycan synthesis arrest, not secondary membrane disruption—a distinction critical for avoiding off-target toxicity [41–43].

Notably, for Category B bioterrorism agents like *Brucella* [44], RS 17053’s dual extracellular/intracellular efficacy surpasses first-line RFP-GM combinations that show high relapse rates [33]. Similarly, its efficacy against MRSA challenges the dogma that PBP2a (MecA) confers broad β-lactam resistance [5,45]. By binding MecA’s allosteric site (GLU-380), it circumvents mutations in the β-lactam interaction domain—a vulnerability exploited by recent covalent inhibitors [46,47]. MecC-MRSA has been increasingly recognized as an emerging zoonotic threat, particularly in livestock and wildlife reservoirs, underscoring the clinical relevance of evaluating novel antibiotics against this variant [48]. Regarding livestock-associated MRSA carrying *mecC*, a homologue of *mecA* sharing approximately 70% amino acid identity, our sequence alignment indicates that the critical binding residue identified in MecA (Glu-380) is conserved in MecC (Fig Z in S File). We therefore predict that RS 17053 would retain activity against *mecC*-MRSA, although direct testing of these strains, which are not routinely available in our laboratory, remains an important direction for future investigation. Its 100% survival rate in bacteremia models (Fig 3I) and rapid abscess resolution (Fig 3K) outperform clinical erythromycin benchmarks—addressing urgent needs for skin/soft tissue infection therapies where failure rates exceed 30% [49]. RS 17053 maintains potent and stable activity under high-inoculum, serum-containing, and cold-storage conditions, with only limited loss of efficacy after extended exposure to body temperature. Furthermore, preliminary testing of RS 17053 against selected highly resistant, *qnr*-positive isolates (e.g., GD-29) showed promising *in vitro* activity (MIC = 32 µg/mL) despite their extensive beta-lactam resistance (AMP MIC > 512 µg/mL). This underscores the necessity to further develop and optimize RS 17053 as a novel therapeutic agent against beta-lactam-resistant bacteria.

Preliminary in silico analysis suggests the potential for structural optimization of the RS 17053 scaffold to further improve binding affinity and predicted toxicity profiles (Fig V in S1 File), highlighting a promising avenue for future lead compound development [50,51]. While this work presents RS 17053 as a promising therapeutic candidate, but the pharmacokinetic and comprehensive safety profile of RS 17053 requires further definition. Efficacy was demonstrated in acute infection models; validation in chronic infection settings and other species is needed. Although our multi-omics data strongly support PBP targeting, the precise structural mechanism awaits future crystallography studies. Finally, the optimized derivatives require empirical synthesis and validation. Addressing these points will be crucial for the compound’s translational advancement.

In conclusion, RS 17053 redefines therapeutic possibilities against intracellular zoonoses. Its triple action—penetrating host cells, evading resistance, and collapsing peptidoglycan biosynthesis—addresses decades-old limitations of β-lactams [7]. As AMR escalates globally [52], leveraging such mechanistically innovative compounds becomes imperative.

## Supporting information

S1 File Fig A.
Class A PBP is the core for the survival and pathogenicity of *Brucella*. This figure shows the cell wall membrane structure of *Brucella* (including outer membrane, peptidoglycan layer, and inner membrane) and its key virulence components (lipid A, lipopolysaccharides, porins, surface proteins, etc.). The key regulatory role of Class A PBPs in the bacterial lifecycle was highlighted. Class A PBP is a core molecular hub that connects the maintenance of *Brucella* structure, intracellular adaptation, and pathogenic processes. Created in BioRender. Tu, D. (2026) https://BioRender.com/zgkibv0. **Fig B. Molecular structure and information of RS 17053. Fig C. SDS-PAGE analysis of target proteins of RS 17053.** (A) The expression of MrcA was analysed by uncropped and unprocessed of SDS-PAGE. The expected size of MrcA is about 37.5 kDa. (B) The expression of MecA was analysed by uncropped and unprocessed of SDS-PAGE. The expected size of MecA is about 70 kDa. (C) The expression of MrcA and MecA after amino acid point mutations was analysed by uncropped and unprocessed of SDS-PAGE. The expected size of mutated MrcA is about 37.5 kDa and mutated MecA is about 70 kDa, respectively. **Fig D. Maximum inhibitory diameter of RS 17053 against various bacterial strains.** The drug loading of RS 17053 on each drug sensitive tablet is 45 μg. The drug loading of quality control drug GM on each drug sensitive paper is 120 μg. Analyze the results according to CLSI’s criteria. Three sets of biological replicates. **Fig E. Observation of the ability of different concentrations compounds or drugs to clear *B. melitensis* TZ in RAW264.7 cells at 24 hours under the confocal laser microscope.** Cells appear blue; *B. melitensis* TZ appear green. Scale bar, 100 μm. **Fig F. Observation of the dynamic lysis process of *B. melitensis* TZ by transmission electron microscopy (TEM) under MIC inhibitory concentration of Ampicillin.** The yellow dashed box highlight the compromised cell wall membrane structure. Scale bar, 500 nm. **Fig G. Acute toxicity of RS 17053 on BALB/c mice.** (A) Survival rates of BALB/c mice at different dosages of RS 17053 were (n = 6, mice). (B) Gross autopsy of mice spleen. Scale bar, 0.5 cm. (C) Concentration of Alkaline phosphatase (AKP), mouse Interleukin-1 (IL-1), mouse Interleukin-6 (IL-6) and mouse Tumor Necrosis Factor-alpha (TNF-α) in serum (n = 5, mice). **Fig H. *In vivo* treatment schematic diagram of RS 17053 on *B. melitensis* 16M infection in BALB/c mice (n = 6, mice).** After 5 days of adaptive feeding, the mice were infected with bacteria, and on the first day of infection, they were continuously administered by intramuscular injection at a dose of 10mg/kg/day for 5 days. On the 15th day of infection, all mice were euthanized and various indicators were tested. Created in BioRender. Tu, D. (2026) https://BioRender.com/zgkibv0. **Fig I. Histopathological assessment of the livers and spleens in different treatment groups in a mouse model of *S. typhimurium* SL1344 infection.** Scale bar of livers, 50 μm; Scale bar of spleens, 100 μm. **Fig J. Gross autopsy of livers, spleens and kidneys of mice infected by *S. typhimurium* SL1344 and MRSA.** (A) *S. typhimurium* SL1344. (B) MRSA. **Fig K. Concentration of AKP, mouse IL-1, IL-6, mouse TNF-α, percentage of lymphocytes (Lymph %) and percentage of monocytes (Mon%) in serum.** (n = 3, mice). **Fig L. Transcriptomic analysis of *B. melitensis TZ* post incubation with RS 17053.** (A) GO annotation analysis. (B) GO annotation enrichment analysis. (C) KEGG annotation analysis. (D) KEGG enrichment analysis. (E) Clustering heatmap of some important DEGs. (F) GO functional enrichment chord diagram. **Fig M. Molecular Dynamics Simulation of RS 17053 and MecA.** (A-D) RMSD, RMSF, Rg and H-bonds number of RS 17053 and the MrcA complexes in a 100 ns MD simulation. **Fig N. Proteomic analysis of MRSA ATCC43300 post incubation with RS 17053.** (A) GO annotation analysis. (B) GO annotation enrichment analysis. (C) KEGG annotation analysis. (D) KEGG enrichment analysis. (E) Clustering heatmap of some important DEGs. (F) KEGG pathway enrichment chord diagram. **Fig O. Transcriptomic analysis of MRSA ATCC43300 post incubation with RS 17053.** (A) GO annotation analysis. (B) GO annotation enrichment analysis. (C) KEGG annotation analysis. (D) KEGG enrichment analysis. (E) Clustering heatmap of some important DEGs. (F) KEGG pathway enrichment chord diagram. **Fig P. Peptidoglycan biosynthesis pathway of the *B. melitensis* TZ, with blue box indicating downregulation.** Red dash circle indicates indispensable biological process. **Fig Q. Peptidoglycan biosynthesis pathway of the MRSA, with blue box indicating downregulation.** Red dash circle indicates indispensable biological process. **Fig R. RT-qPCR to verify differentially expressed genes of *B. melitensis* TZ and MRSA in the presence of RS 17053.** MRSA ATCC 43300 was treated with RS 17053 at 4 × MIC for 3 h. Relative expression of *mecA* (primary target) and *blaZ* (β-lactamase) was determined, with EF-Tu as internal control. Data are mean ± SD (n = 3 biological replicates). **Fig S. Western blot to verify the binding targets discovered in multiple omics. Fig T. Class A PBPs (MrcA and MecA) are essential proteins involved in the GTase and TPase stages of peptidoglycan biosynthesis.** Created in BioRender. Tu, D. (2026) https://BioRender.com/zgkibv0. **Fig U. MRSA ATCC43300 primary genome-wide assembly map. Fig V. Skeleton optimization of RS 17053 based on chemical experts.** (A) Chemdraw version 20.0 drew derivatives of RS 17053. (B) Thermograms of binding energies for the top 9 best binding models of different newly modified compounds and MrcA protein. (C) Thermograms of binding energies for the top 9 best binding models of different newly modified compounds and MecA protein. **Fig W. Changes in bacterial ATP and ROS levels under RS 17053 treatment. Fig X. Sensitivity changes of *B.suis ΔmrcA* S2 to RS 17053.** (A) PCR amplification of *mrcA* gene upstream homologous arm (897 bp), downstream homologous arm (896 bp), and pSK2 empty vector (4516 bp). (B) Verify the effect of multi fragment ligation transformation through PCR. (C) PCR verification of *mrcA* gene deletion. (D) Detection of resistance phenotype of *B. suis ΔmrcA* S2 to RS 17053 **Fig Y. The influence of different physiological conditions on the sensitivity of RS 17053.** (A) The effect of different inoculation amounts on the sensitivity of RS 17053. (B) The effect of different culture medium formulations on the sensitivity of RS 17053. (C, D) The effect of different storage temperatures on the sensitivity of RS 17053. **Fig Z. Homology alignment and conservation analysis of MecA and MecC amino acid sequences.**
(DOCX)

S1 Table 150 compounds with potential antimicrobial effects.
(DOCX)

S2 Table Mouse Blood Pressure Measurements (mmHg).
(DOCX)

S3 Table Broad-spectrum antimicrobial activity of RS 17053.
(DOCX)

S4 Table RT-qPCR assays primers.
(DOCX)

S5 Table Combined raw data (Excel file).
(XLSX)
