# Supplementary material for: A novel penicillin-binding protein inhibitor with unprecedented intracellular activity eradicates multiple pathogenic bacteria
Source: PLoS Pathog. 2026 Jul 16;22(7):e1014242. doi: 10.1371/journal.ppat.1014242 (PMC13374901; doi:10.1371/journal.ppat.1014242)
Supplement: S1 File — Fig A. Class A PBP is the core for the survival and pathogenicity of Brucella. This figure shows the cell wall membrane structure of Brucella (including outer membrane, peptidoglycan layer, and inner membrane) and its key virulence components (lipid A, lipopolysaccharides, porins, surface proteins, etc.). The key regulatory role of Class A PBPs in the bacterial lifecycle was highlighted. Class A PBP is a core molecular hub that connects the maintenance of Brucella structure, intracellular adaptation, and pathogenic processes. Created in BioRender. Tu, D. (2026) https://BioRender.com/zgkibv0. Fig B. Molecular structure and information of RS 17053. Fig C. SDS-PAGE analysis of target proteins of RS 17053. (A) The expression of MrcA was analysed by uncropped and unprocessed of SDS-PAGE. The expected size of MrcA is about 37.5 kDa. (B) The expression of MecA was analysed by uncropped and unprocessed of SDS-PAGE. The expected size of MecA is about 70 kDa. (C) The expression of MrcA and MecA after amino acid point mutations was analysed by uncropped and unprocessed of SDS-PAGE. The expected size of mutated MrcA is about 37.5 kDa and mutated MecA is about 70 kDa, respectively. Fig D. Maximum inhibitory diameter of RS 17053 against various bacterial strains. The drug loading of RS 17053 on each drug sensitive tablet is 45 μg. The drug loading of quality control drug GM on each drug sensitive paper is 120 μg. Analyze the results according to CLSI’s criteria. Three sets of biological replicates. Fig E. Observation of the ability of different concentrations compounds or drugs to clear B. melitensis TZ in RAW264.7 cells at 24 hours under the confocal laser microscope. Cells appear blue; B. melitensis TZ appear green. Scale bar, 100 μm. Fig F. Observation of the dynamic lysis process of B. melitensis TZ by transmission electron microscopy (TEM) under MIC inhibitory concentration of Ampicillin. The yellow dashed box highlight the compromised cell wall membrane structure. Scale bar [file ppat.1014242.s001.docx]

**S1 File. Supporting Information**


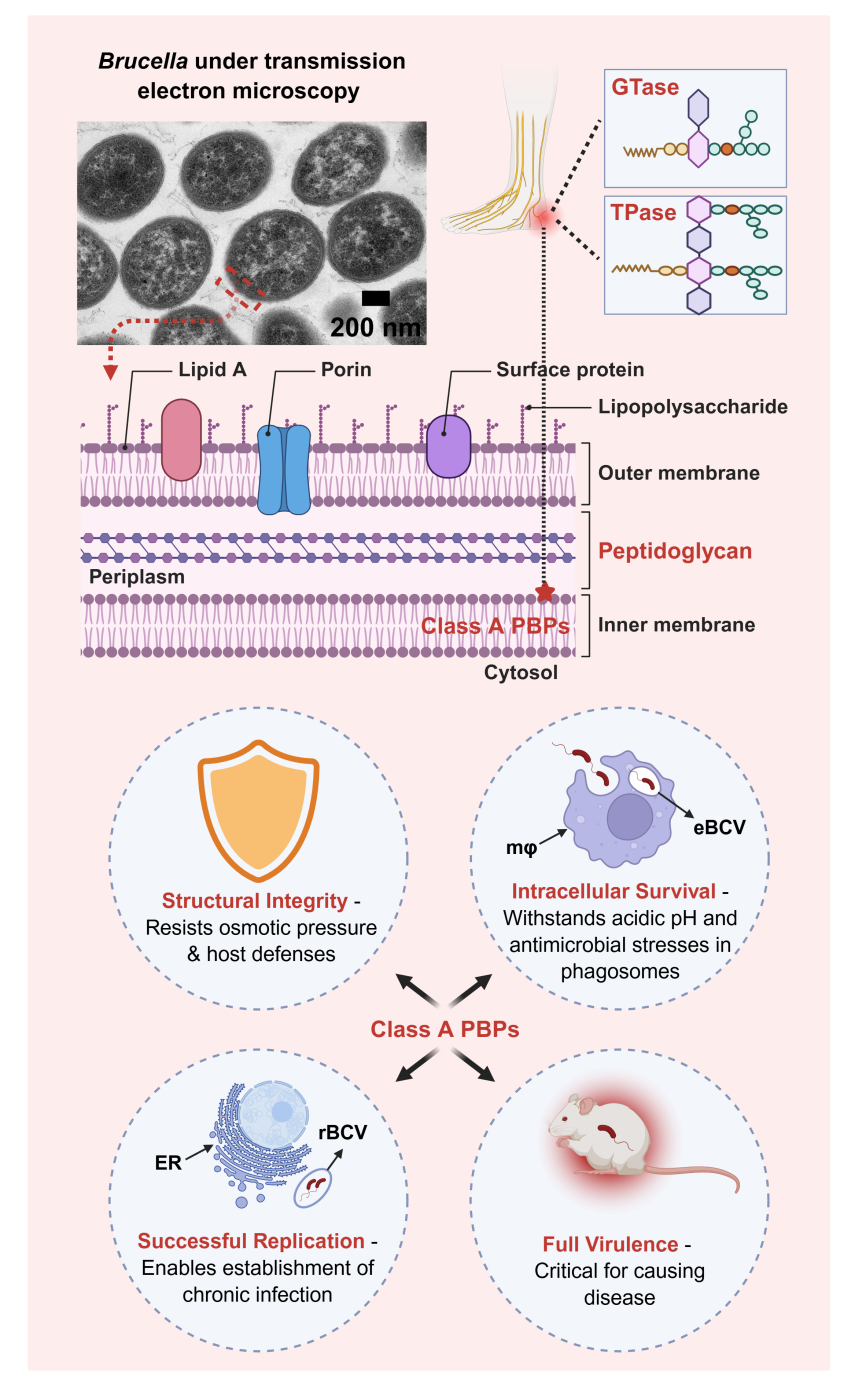


**Fig A. Class A PBP is the core for the survival and pathogenicity of *Brucella*.** This figure shows the cell wall membrane structure of *Brucella* (including outer membrane, peptidoglycan layer, and inner membrane) and its key virulence components (lipid A, lipopolysaccharides, porins, surface proteins, etc.). The key regulatory role of Class A PBPs in the bacterial lifecycle was highlighted. Class A PBP is a core molecular hub that connects the maintenance of *Brucella* structure, intracellular adaptation, and pathogenic processes. Created in BioRender. Tu, D. (2026) https://BioRender.com/zgkibv0.

**
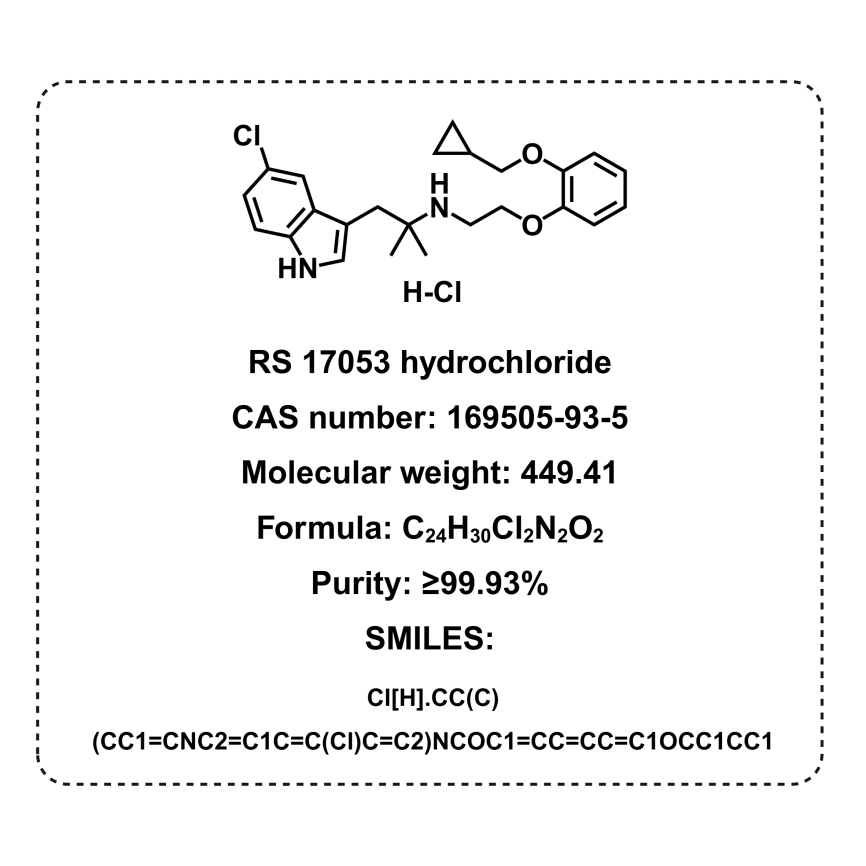
**

**Fig B. Molecular structure and information of RS 17053.**


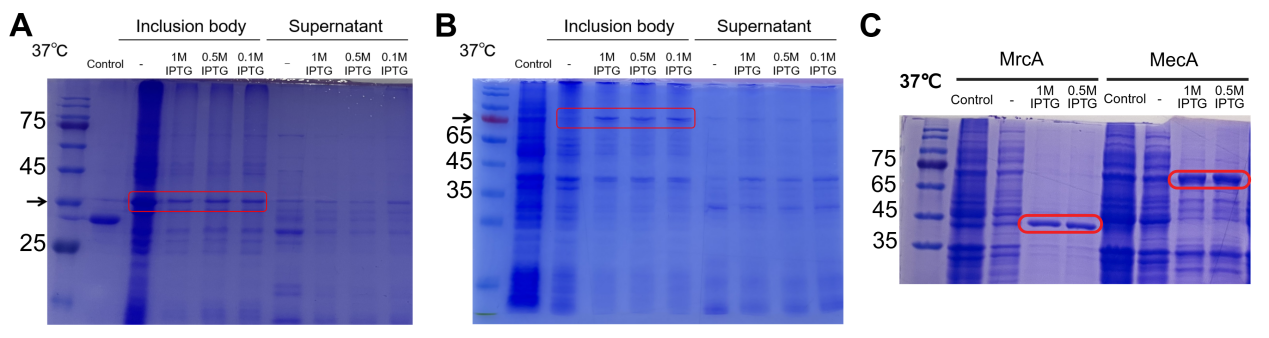


**Fig C. SDS-PAGE analysis of target proteins of RS 17053.** (A) The expression of MrcA was analysed by uncropped and unprocessed of SDS-PAGE. The expected size of MrcA is about 37.5 kDa. (B) The expression of MecA was analysed by uncropped and unprocessed of SDS-PAGE. The expected size of MecA is about 70 kDa. (C) The expression of MrcA and MecA after amino acid point mutations was analysed by uncropped and unprocessed of SDS-PAGE. The expected size of mutated MrcA is about 37.5 kDa and mutated MecA is about 70 kDa, respectively.


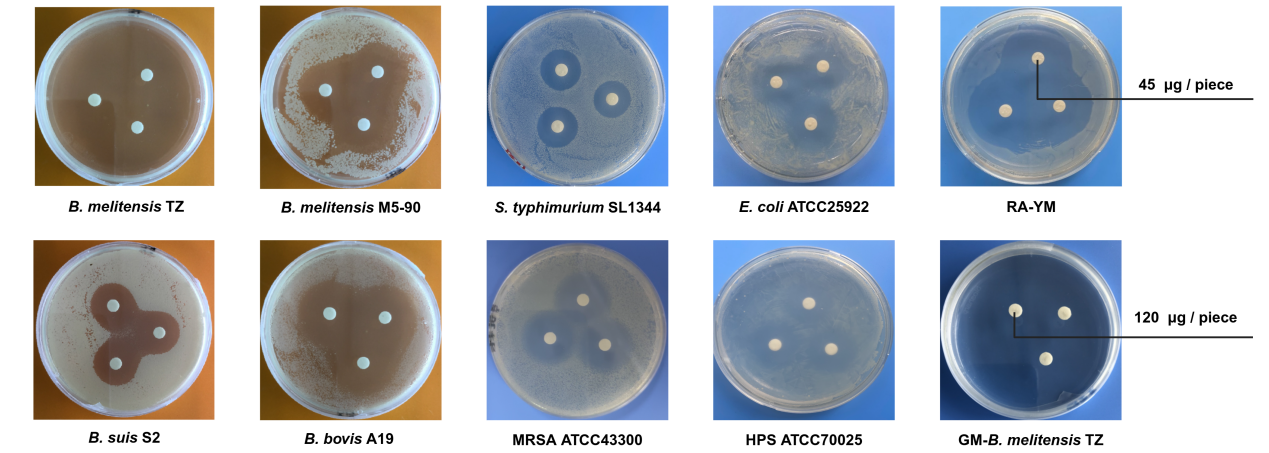


**Fig D. Maximum inhibitory diameter of RS 17053 against various bacterial strains.** The drug loading of RS 17053 on each drug sensitive tablet is 45 μg. The drug loading of quality control drug GM on each drug sensitive paper is 120 μg. Analyze the results according to CLSI's criteria. Three sets of biological replicates.


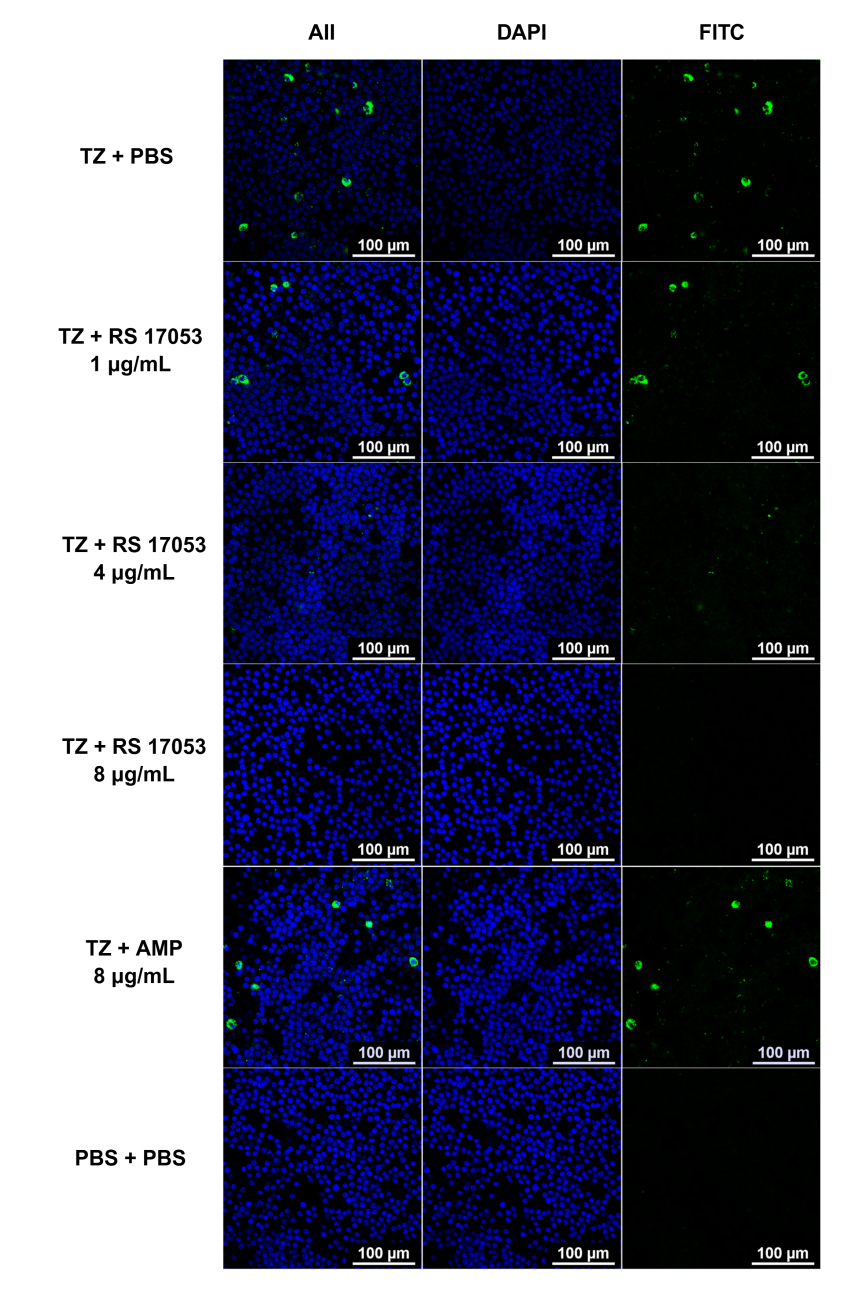


**Fig E. Observation of the ability of different concentrations compounds or drugs to clear *B. melitensis* TZ in RAW264.7 cells at 24 hours under the confocal laser microscope.** Cells appear blue; *B. melitensis* TZ appear green. Scale bar, 100 μm.


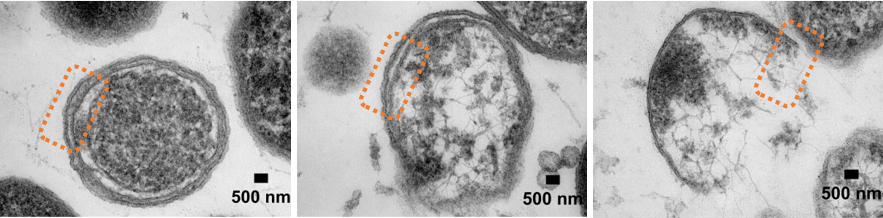


**Fig F. Observation of the dynamic lysis process of *B. melitensis* TZ by transmission electron microscopy (TEM) under MIC inhibitory concentration of Ampicillin.** The yellow dashed box highlight the compromised cell wall membrane structure. Scale bar, 500 nm.


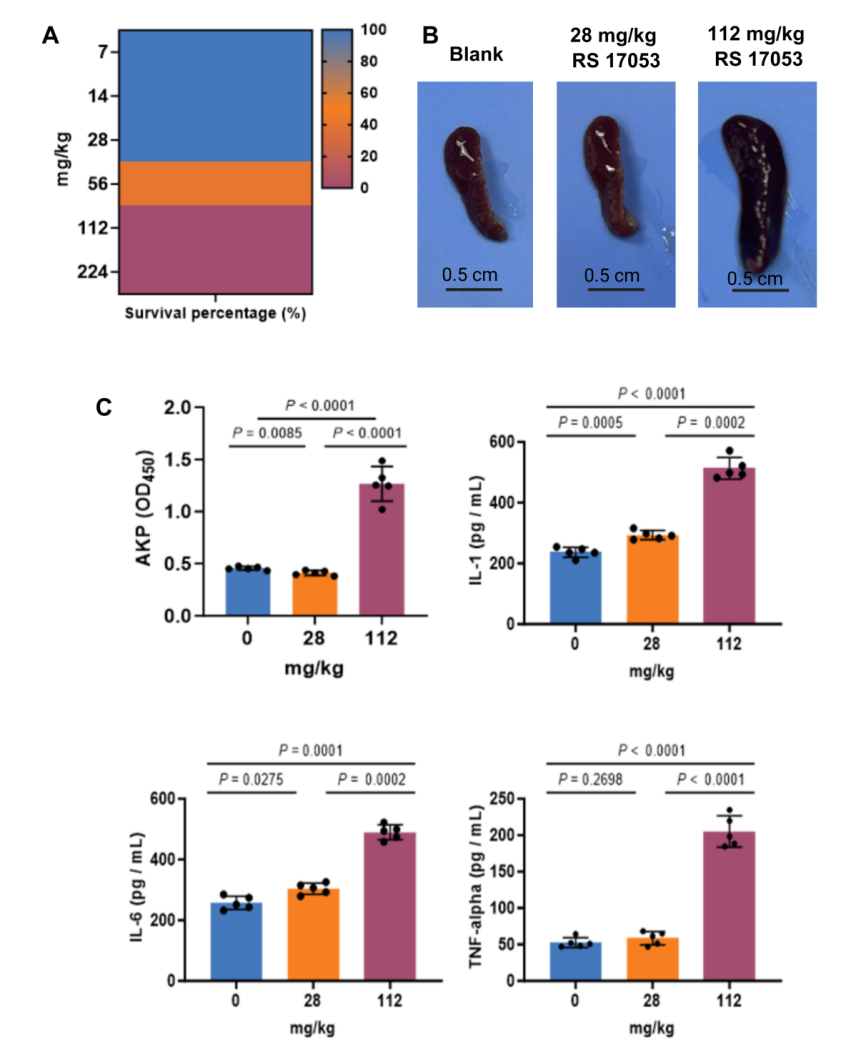


**Fig G. Acute toxicity of RS 17053 on BALB/c mice.** (A) Survival rates of BALB/c mice at different dosages of RS 17053 were (n = 6, mice). (B) Gross autopsy of mice spleen. Scale bar, 0.5 cm. (C) Concentration of Alkaline phosphatase (AKP), mouse Interleukin-1 (IL-1), mouse Interleukin-6 (IL-6) and mouse Tumor Necrosis Factor-alpha (TNF-α) in serum (n = 5, mice).


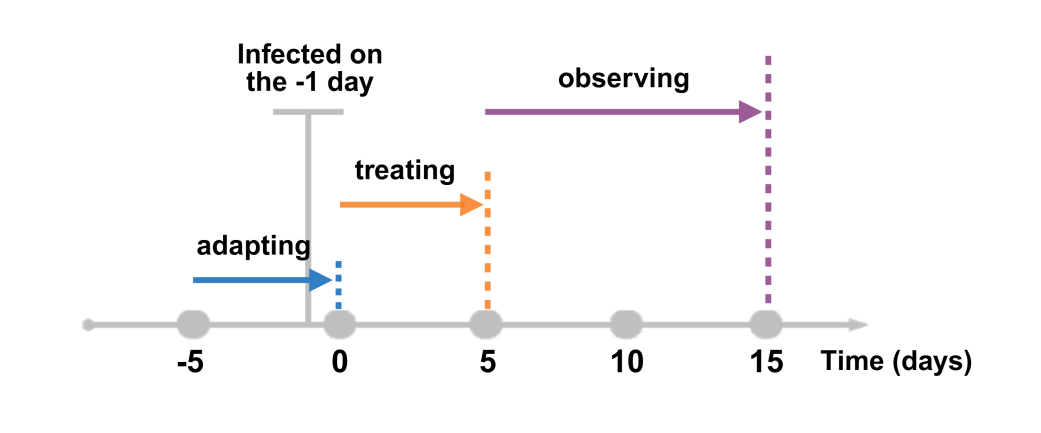


**Fig H. *In vivo* treatment schematic diagram of RS 17053 on *B. melitensis* 16M infection in BALB/c mice (n = 6, mice).** After 5 days of adaptive feeding, the mice were infected with bacteria, and on the first day of infection, they were continuously administered by intramuscular injection at a dose of 10mg/kg/day for 5 days. On the 15th day of infection, all mice were euthanized and various indicators were tested. Created in BioRender. Tu, D. (2026) https://BioRender.com/zgkibv0.


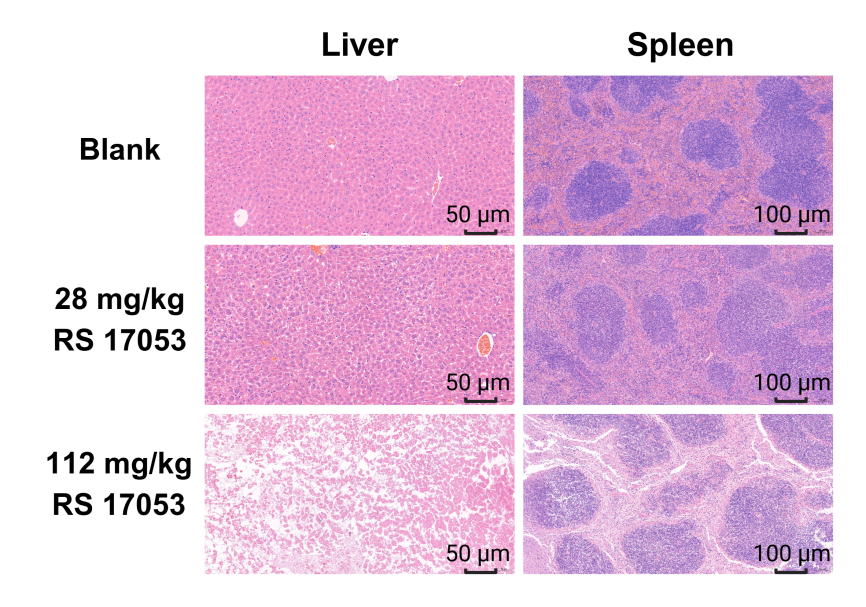


**Fig I. Histopathological assessment of the livers and spleens in different treatment groups in a mouse model of *S. typhimurium* SL1344 infection.** Scale bar of livers, 50 μm; Scale bar of spleens, 100 μm.


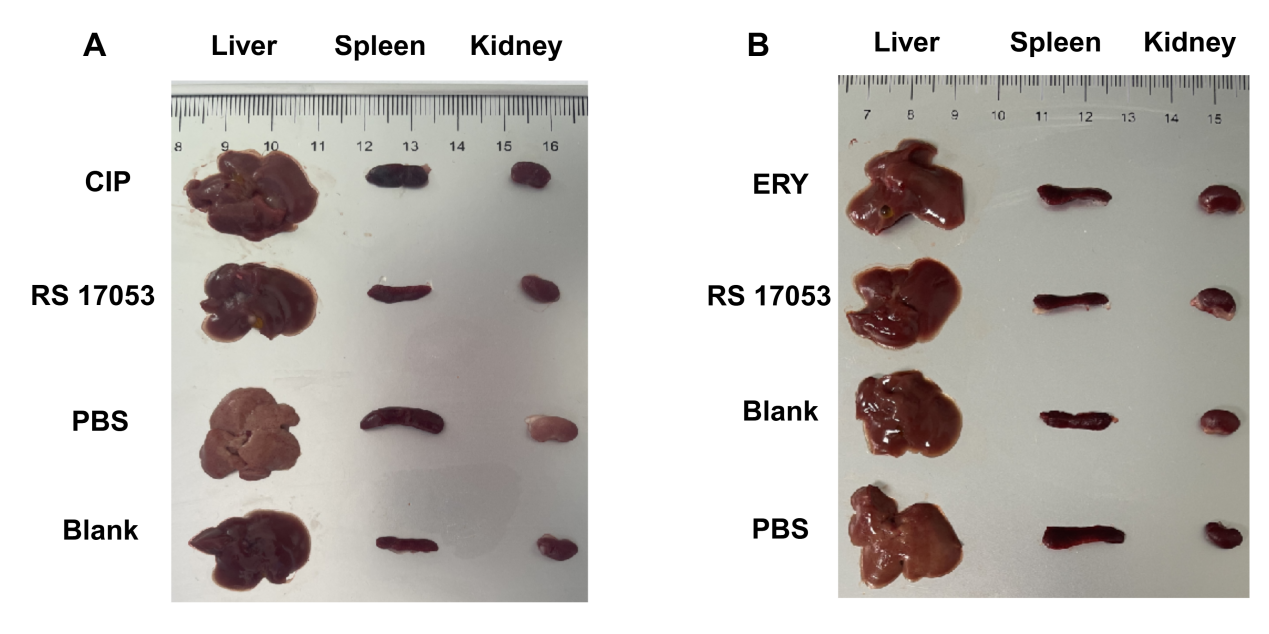


**Fig J. Gross autopsy of livers, spleens and kidneys of mice infected by *S. typhimurium* SL1344 and MRSA.** (A) *S. typhimurium* SL1344. (B) MRSA.


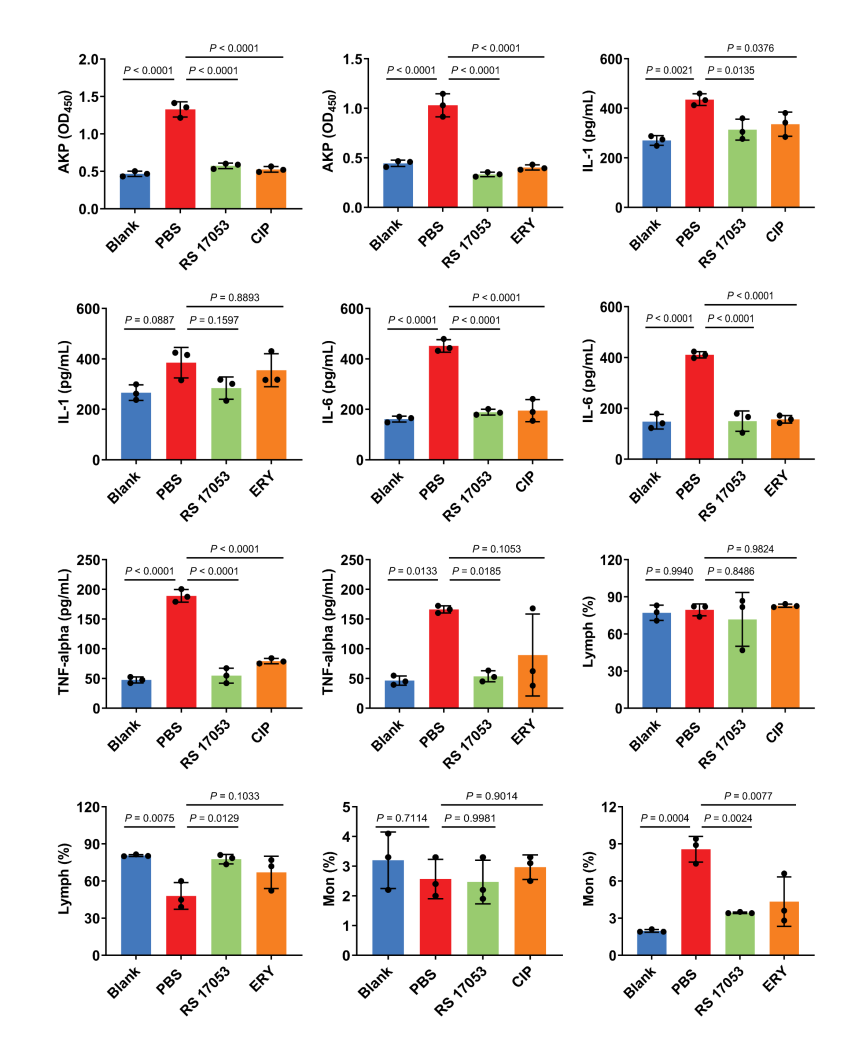


**Fig K. Concentration of AKP, mouse IL-1, IL-6, mouse TNF-α, percentage of lymphocytes (Lymph %) and percentage of monocytes (Mon%) in serum.** (n = 3, mice).


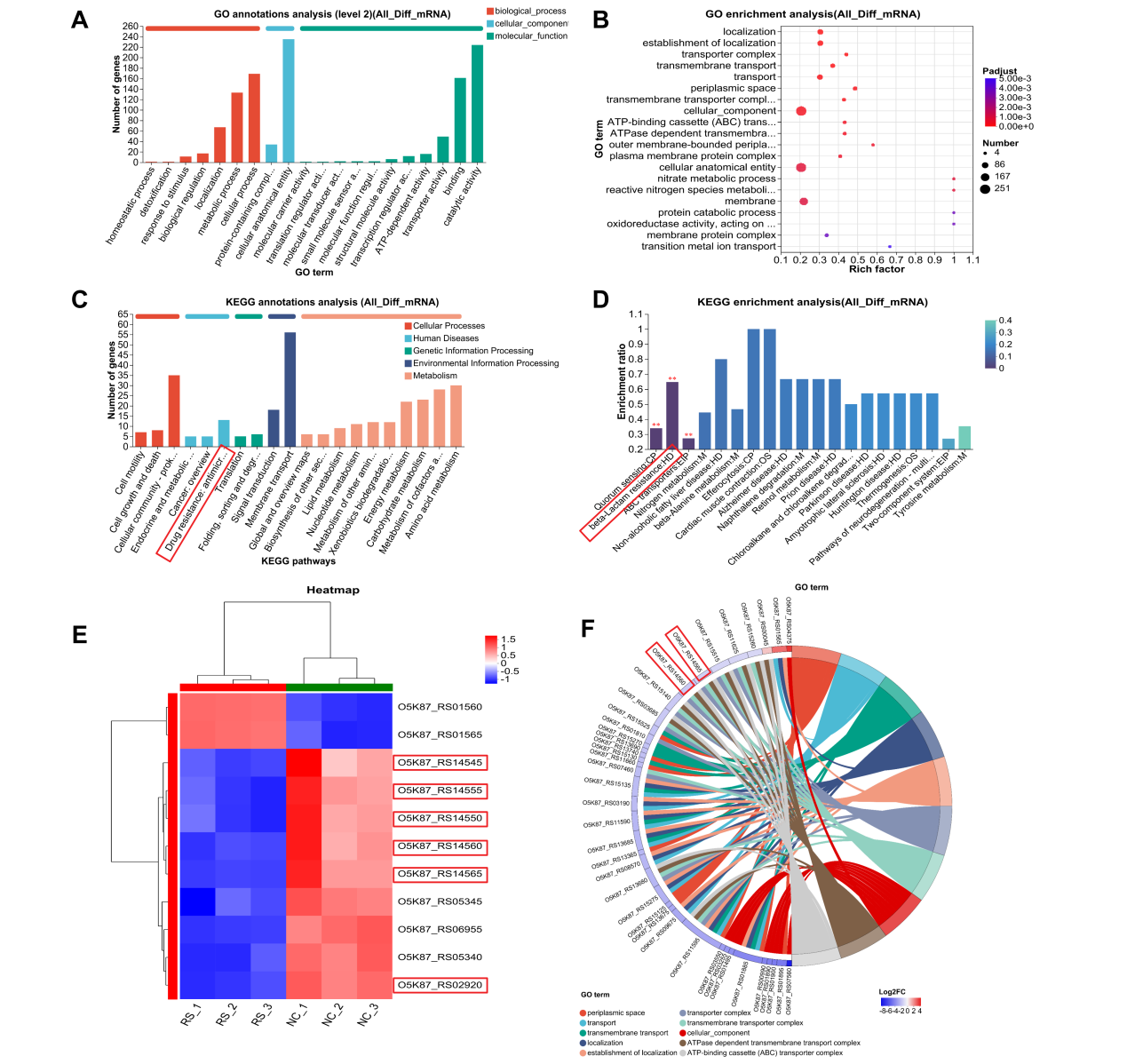


**Fig L. Transcriptomic analysis of *B. melitensis TZ* post incubation with RS 17053.** (A) GO annotation analysis. (B) GO annotation enrichment analysis. (C) KEGG annotation analysis. (D) KEGG enrichment analysis. (E) Clustering heatmap of some important DEGs. (F) GO functional enrichment chord diagram.


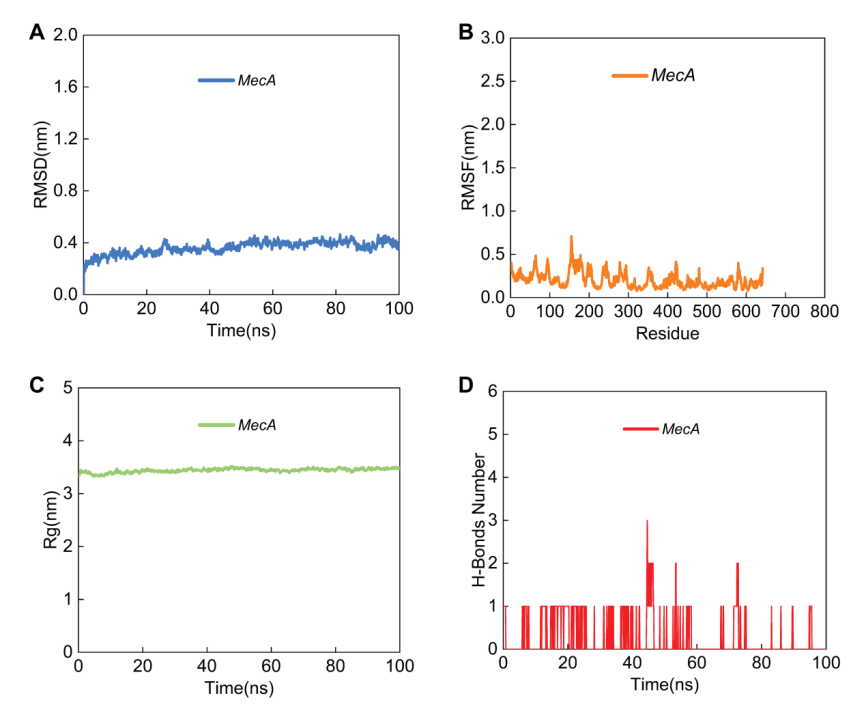


**Fig M. Molecular Dynamics Simulation of RS 17053 and MecA.** (A-D) RMSD, RMSF, Rg and H-bonds number of RS 17053 and the MrcA complexes in a 100 ns MD simulation.


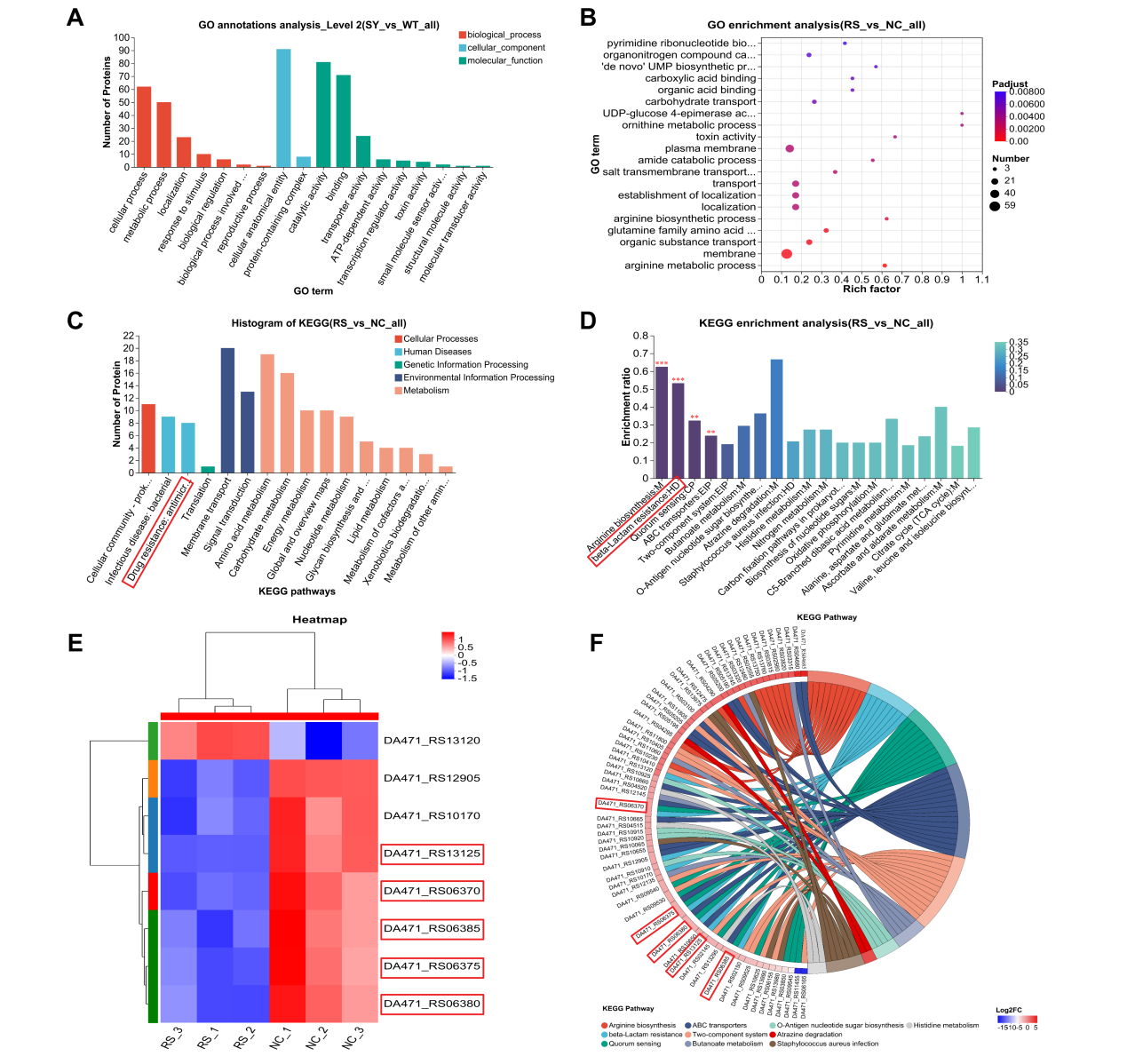


**Fig N. Proteomic analysis of MRSA ATCC43300 post incubation with RS 17053.** (A) GO annotation analysis. (B) GO annotation enrichment analysis. (C) KEGG annotation analysis. (D) KEGG enrichment analysis. (E) Clustering heatmap of some important DEGs. (F) KEGG pathway enrichment chord diagram.


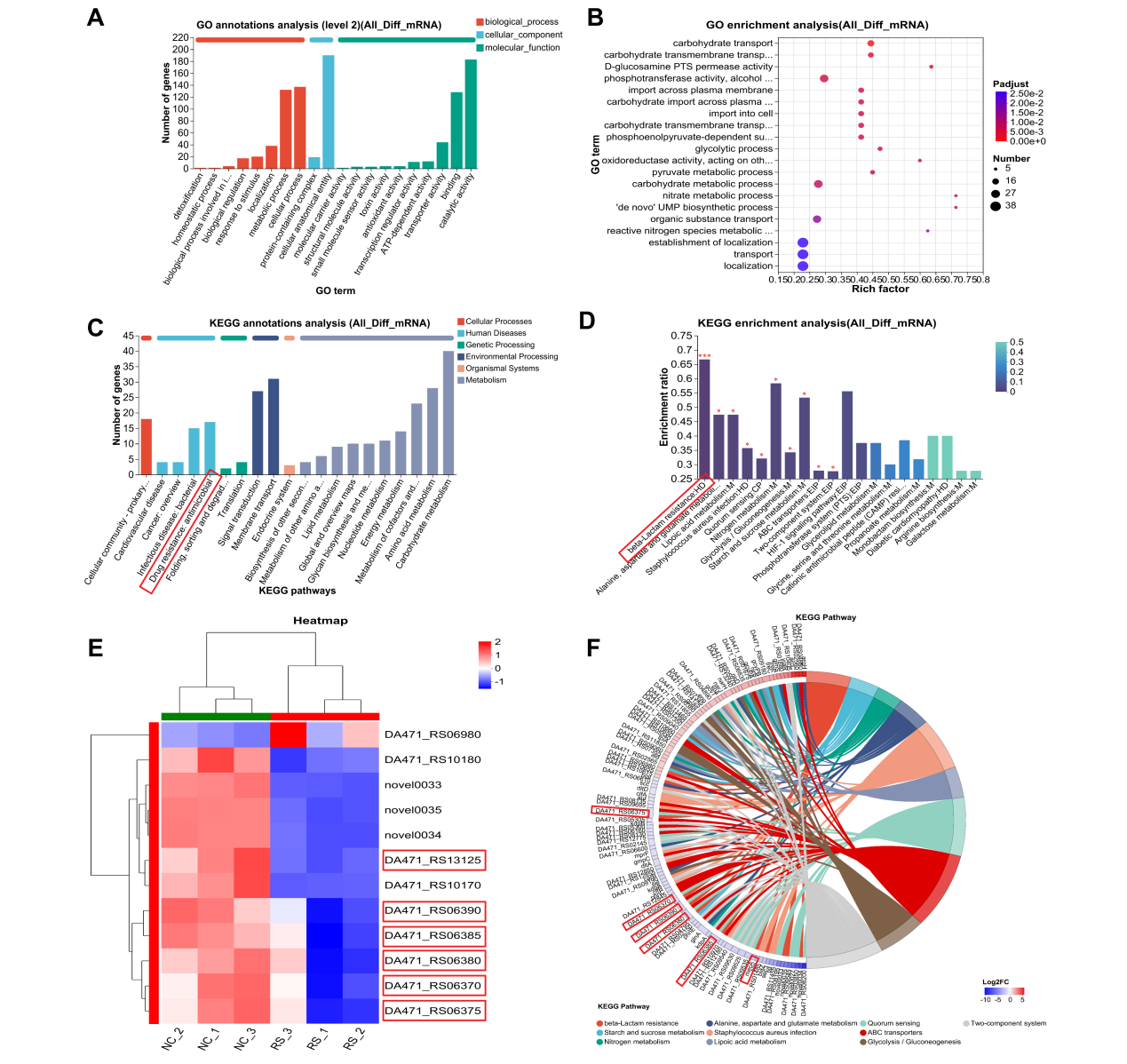


**Fig O. Transcriptomic analysis of MRSA ATCC43300 post incubation with RS 17053.** (A) GO annotation analysis. (B) GO annotation enrichment analysis. (C) KEGG annotation analysis. (D) KEGG enrichment analysis. (E) Clustering heatmap of some important DEGs. (F) KEGG pathway enrichment chord diagram.


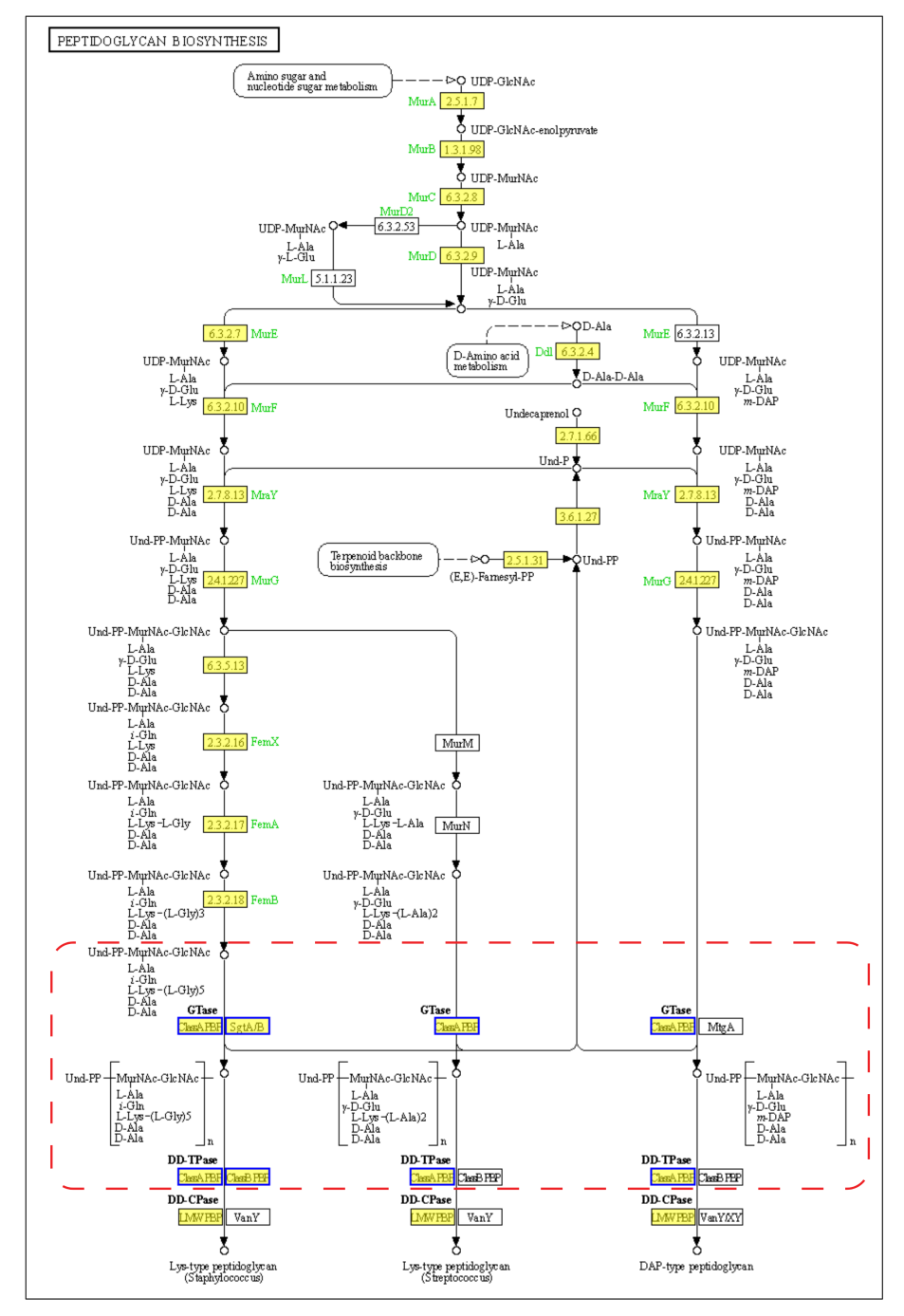


**Fig P. Peptidoglycan biosynthesis pathway of the *B. melitensis* TZ, with blue box indicating downregulation.** Red dash circle indicates indispensable biological process.


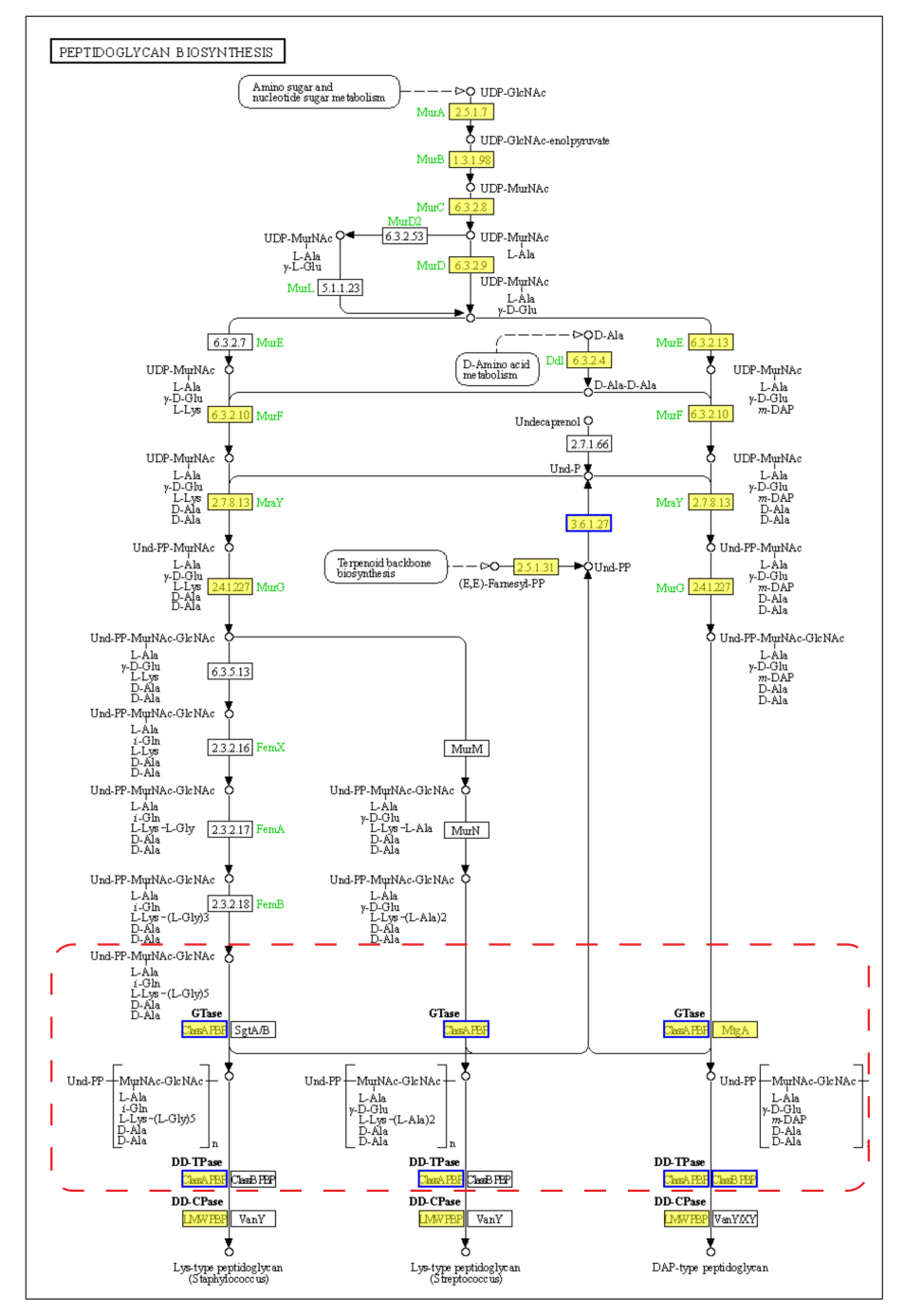


**Fig Q. Peptidoglycan biosynthesis pathway of the MRSA, with blue box indicating downregulation.** Red dash circle indicates indispensable biological process.


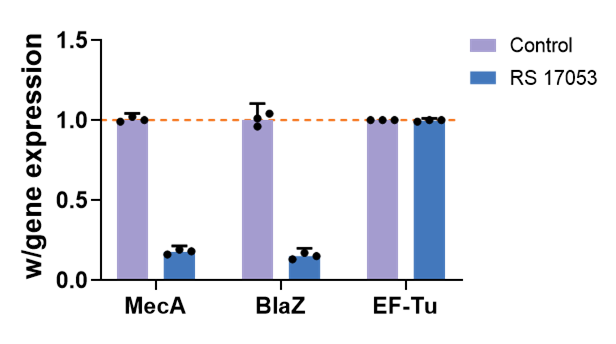


**Fig R. RT-qPCR to verify differentially expressed genes of *B. melitensis* TZ and MRSA in the presence of RS 17053.** MRSA ATCC 43300 was treated with RS 17053 at 4×MIC for 3 h. Relative expression of *mecA* (primary target) and *blaZ* (β-lactamase) was determined, with EF-Tu as internal control. Data are mean ± SD (n=3 biological replicates).


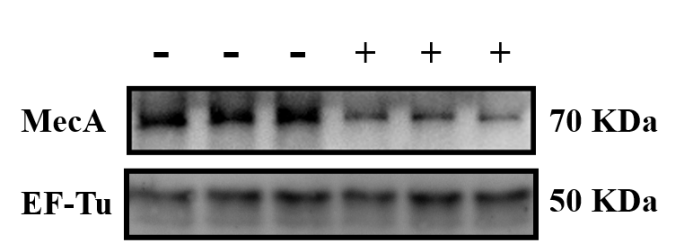


**Fig S. Western blot to verify the binding targets discovered in multiple omics.**

**
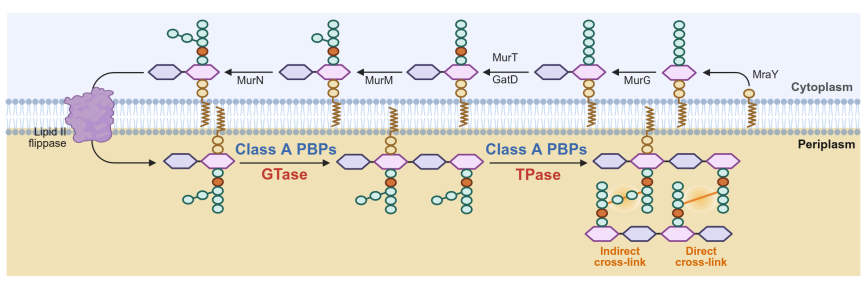
**

**Fig T. Class A PBPs (MrcA and MecA) are essential proteins involved in the GTase and TPase stages of peptidoglycan biosynthesis.** Created in BioRender. Tu, D. (2026) https://BioRender.com/zgkibv0.


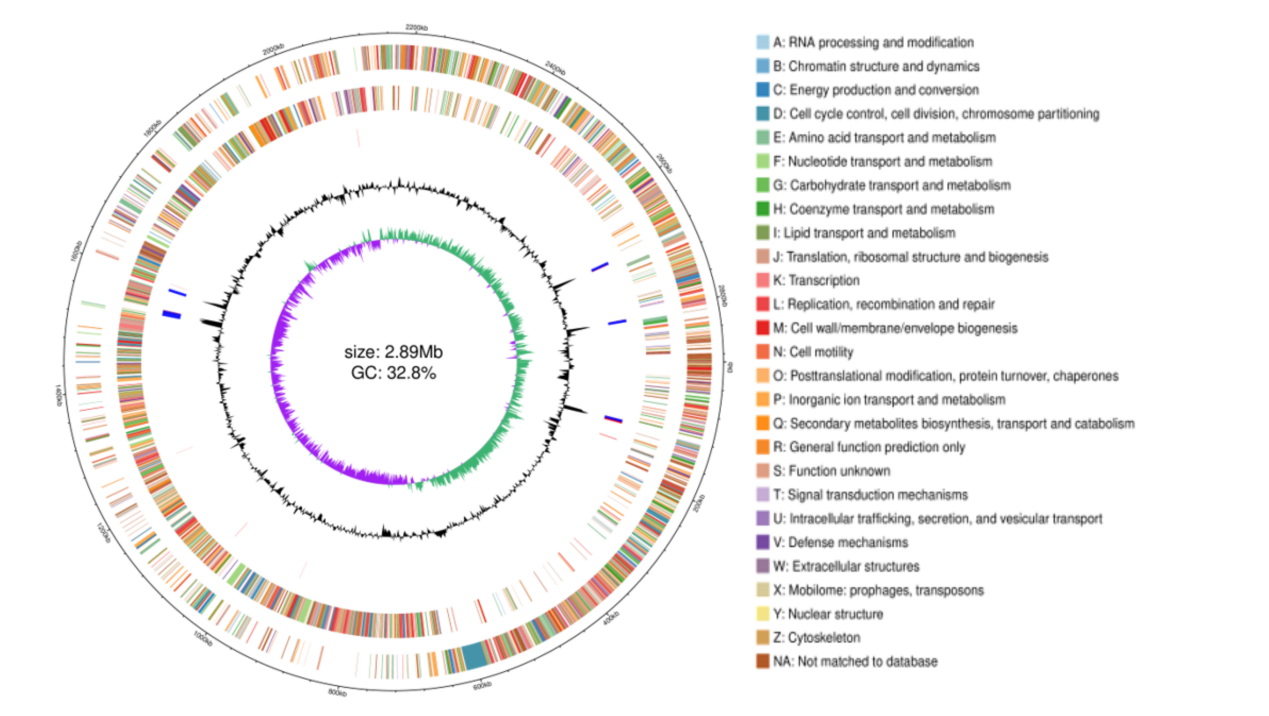


**Fig U. MRSA ATCC43300 primary genome-wide assembly map.**


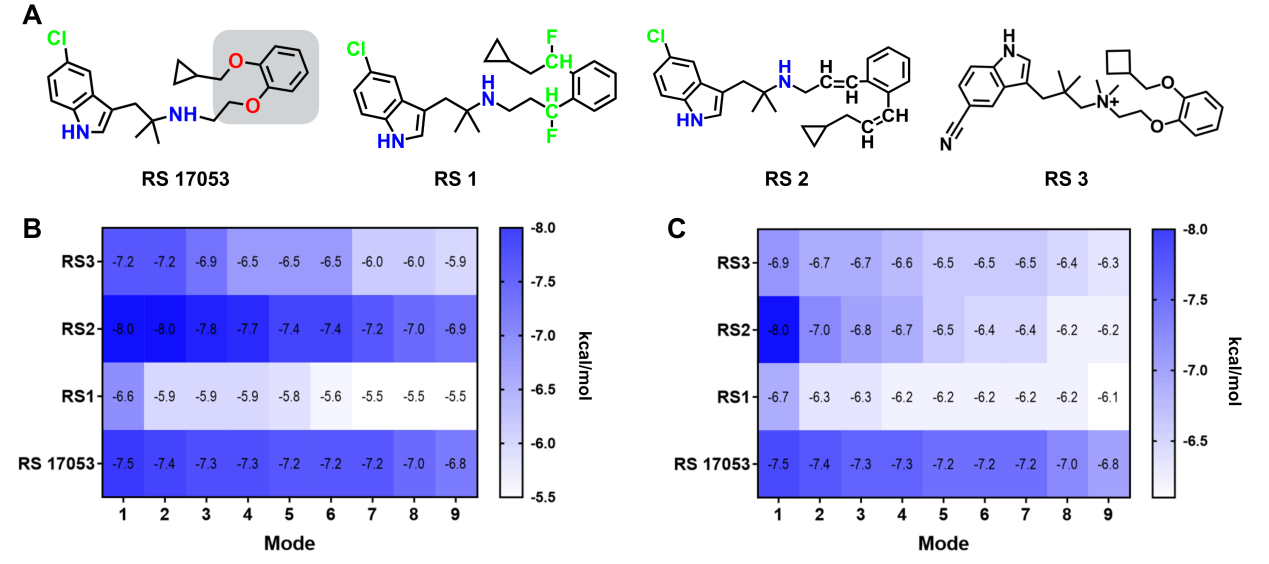


**Fig V. Skeleton optimization of RS 17053 based on chemical experts.** (A) Chemdraw version 20.0 drew derivatives of RS 17053. (B) Thermograms of binding energies for the top 9 best binding models of different newly modified compounds and MrcA protein. (C) Thermograms of binding energies for the top 9 best binding models of different newly modified compounds and MecA protein.


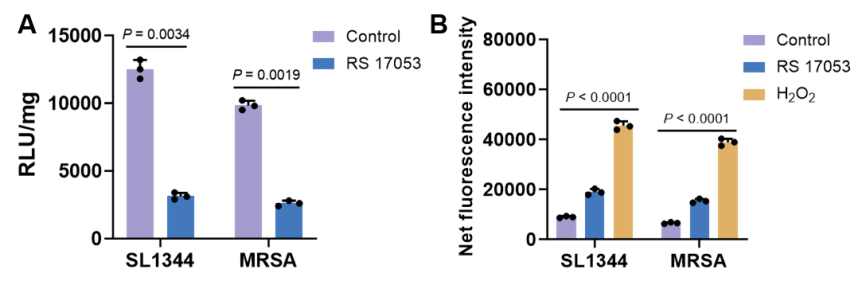


**Fig W. Changes in bacterial ATP and ROS levels under RS 17053 treatment.** (A) ATP. (B) ROS. All data are presented as mean ± SD (n = 3 independent biological replicates). Statistical comparisons were performed using unpaired two-tailed Student’s t-test. Mean differences and 95% confidence intervals (CI) were calculated using the Hedges–Olkin method. Cohen’s d effect sizes are reported in the main text.


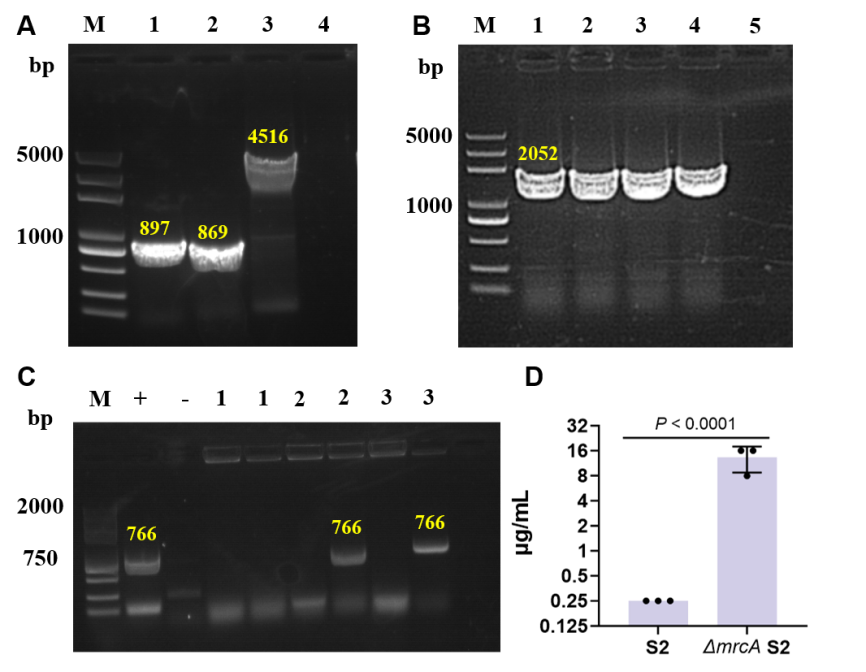


**Fig X. Sensitivity changes of *B.suis ΔmrcA* S2 to RS 17053.** (A) PCR amplification of *mrcA* gene upstream homologous arm (897 bp), downstream homologous arm (896 bp), and pSK2 empty vector (4516 bp). (B) Verify the effect of multi fragment ligation transformation through PCR. (C) PCR verification of *mrcA* gene deletion. (D) Detection of resistance phenotype of *B. suis ΔmrcA* S2 to RS 17053.


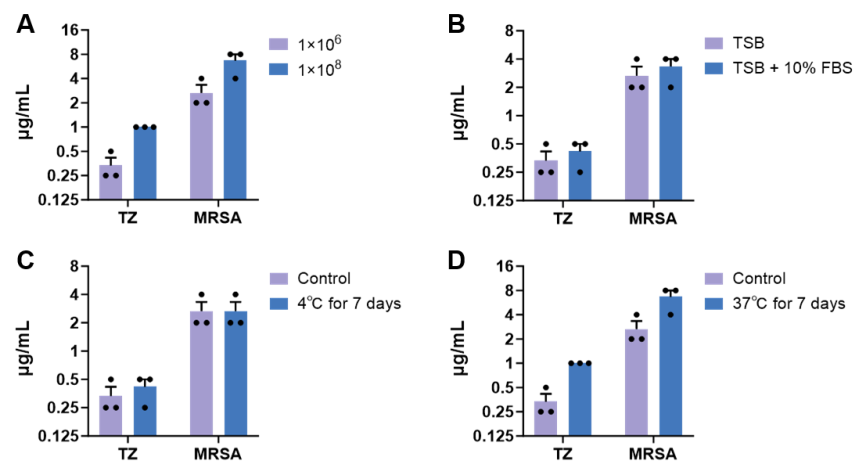


**Fig Y. The influence of different physiological conditions on the sensitivity of RS 17053.** (A) The effect of different inoculation amounts on the sensitivity of RS 17053. (B) The effect of different culture medium formulations on the sensitivity of RS 17053. (C, D) The effect of different storage temperatures on the sensitivity of RS 17053.


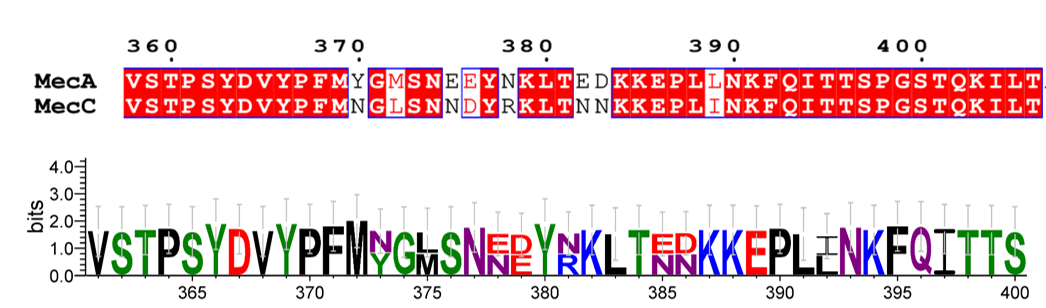


**Fig Z. Homology alignment and conservation analysis of MecA and MecC amino acid sequences.**
