# Supplementary material for: A novel penicillin-binding protein inhibitor with unprecedented intracellular activity eradicates multiple pathogenic bacteria
Source: PLoS Pathog. 2026 Jul 16;22(7):e1014242. doi: 10.1371/journal.ppat.1014242 (PMC13374901; doi:10.1371/journal.ppat.1014242)
Supplement: S1 Table — 150 compounds with potential antimicrobial effects. (DOCX) [file ppat.1014242.s002.docx]

**A novel penicillin-binding protein inhibitor with unprecedented intracellular activity eradicates multiple pathogenic bacteria**

**S1 Table. 150 compounds with potential antimicrobial effects.**

| Item | Catalog No. | Drug Name | CAS No. | Formula | Mw | Clinical Information |
| --- | --- | --- | --- | --- | --- | --- |
| 1 | HY-101336 | RS 17053 hydrochloride | 169505-93-5 | C24H30Cl2N2O2 | 412.95 | No Development Reported |
| 2 | HY-131338 | RORγt inverse agonist 13 | 2170477-75-3 | C23H17Cl2F3N2O4 | 513.29 | No Development Reported |
| 3 | HY-13967B | AMG 837 (calcium hydrate) | 1259389-38-2 | C26H22F3O4- | 438.44 | No Development Reported |
| 4 | HY-126124 | AP39 | 1429061-80-2 | C37H38BrO2PS3 | 641.86 | No Development Reported |
| 5 | HY-131232 | Desmorpholinyl Navitoclax-NH-Me | 2365172-82-1 | C44H51ClF3N5O5S3 | 918.55 | No Development Reported |
| 6 | HY-13467 | AM-1638 | 1142214-62-7 | C33H35FO4 | 514.63 | No Development Reported |
| 7 | HY-134656 | BC1618 | 2222094-18-8 | C24H24F3NO2 | 415.45 | No Development Reported |
| 8 | HY-10627A | GW3965 (hydrochloride) | 405911-17-3 | C33H32Cl2F3NO3 | 582.05 | No Development Reported |
| 9 | HY-100447 | TM5275 (sodium) | 1103926-82-4 | C28H27ClN3NaO5 | 521.99 | No Development Reported |
| 10 | HY-119293 | K777 | 233277-99-1 | C32H38N4O4S | 574.73 | No Development Reported |
| 11 | HY-134832 | Mito-LND | 2361564-49-8 | C43H45BrCl2N3OP | 721.72 | No Development Reported |
| 12 | HY-101398 | Coproporphyrin III | 14643-66-4 | C36H38N4O8 | 654.71 | No Development Reported |
| 13 | HY-131347 | PD-1/PD-L1-IN-NP19 | 2377916-66-8 | C33H31ClN2O4 | 555.06 | No Development Reported |
| 14 | HY-112445 | SGC3027 |  | C41H47ClN6O6S | 787.37 | No Development Reported |
| 15 | HY-13245 | PF-4136309 | 1341224-83-6 | C29H31F3N6O3 | 568.59 | No Development Reported |
| 16 | HY-12291 | HG6-64-1 | 1315329-43-1 | C32H34F3N5O2 | 577.64 | No Development Reported |
| 17 | HY-100747 | PSB-12379 | 1802226-78-3 | C18H23N5O9P2 | 515.35 | No Development Reported |
| 18 | HY-12762 | QS11 | 944328-88-5 | C36H33N5O2 | 567.68 | No Development Reported |
| 19 | HY-106147B | Frakefamide (TFA) |  | C32H35F4N5O7 | 563.62 | No Development Reported |
| 20 | HY-137499 | NT1-O12B |  | C36H60N2O4S4 | 713.13 | No Development Reported |
| 21 | HY-107394 | UK 356618 | 230961-08-7 | C34H43N3O4 | 557.72 | No Development Reported |
| 22 | HY-120072 | PF-3450074 | 1352879-65-2 | C27H27N3O2 | 425.52 | No Development Reported |
| 23 | HY-126320 | EGFR-IN-8 | 2407957-87-1 | C32H23ClF3N7O4 | 662.02 | No Development Reported |
| 24 | HY-124729 | BL-918 | 2101517-69-3 | C23H15F8N3OS | 533.44 | No Development Reported |
| 25 | HY-124729A | (Rac)-BL-918 | 2435589-07-2 | C23H15F8N3OS | 533.44 | No Development Reported |
| 26 | HY-14440 | MP7 | 1001409-50-2 | C28H22F2N4O4 | 516.50 | No Development Reported |
| 27 | HY-12831 | Ampkinone | 1233082-79-5 | C31H23NO6 | 505.52 | No Development Reported |
| 28 | HY-15607A | WEHI-539 hydrochloride | 2070018-33-4 | C31H30ClN5O3S2 | 583.72 | No Development Reported |
| 29 | HY-10425 | A-443654 | 552325-16-3 | C24H23N5O | 397.47 | No Development Reported |
| 30 | HY-101502A | SB290157 (trifluoroacetate) | 1140525-25-2 | C24H29F3N4O6 | 412.48 | No Development Reported |
| 31 | HY-112587 | MC3482 |  | C33H38N4O8 | 618.68 | No Development Reported |
| 32 | HY-123205 | Oxatomide | 60607-34-3 | C27H30N4O | 426.55 | No Development Reported |
| 33 | HY-12759 | CARM1-IN-1 | 1020399-49-8 | C26H21Br2NO3 | 555.26 | No Development Reported |
| 34 | HY-112257 | S-23 | 1010396-29-8 | C18H13ClF4N2O3 | 416.75 | No Development Reported |
| 35 | HY-15529 | S0859 | 1019331-10-2 | C29H24ClN3O3S | 530.04 | No Development Reported |
| 36 | HY-15300 | Skepinone-L | 1221485-83-1 | C24H21F2NO4 | 425.42 | No Development Reported |
| 37 | HY-136241 | OT-82 | 1800487-55-1 | C26H21FN4O | 424.47 | No Development Reported |
| 38 | HY-119254 | BAY-850 | 2099142-76-2 | C38H44ClN5O3 | 654.24 | No Development Reported |
| 39 | HY-12461 | WS6 | 1421227-53-3 | C29H31F3N6O3 | 568.59 | No Development Reported |
| 40 | HY-12812 | Autotaxin modulator 1 | 1548743-69-6 | C28H31F6NO3 | 543.54 | No Development Reported |
| 41 | HY-114395A | (R)-NVS-ZP7-4 |  | C28H28FN5OS | 501.62 | No Development Reported |
| 42 | HY-12280 | THZ2 | 1604810-84-5 | C31H28ClN7O2 | 566.05 | No Development Reported |
| 43 | HY-126428 | ZL0580 | 2377151-10-3 | C25H23F3N4O4S | 532.53 | No Development Reported |
| 44 | HY-15520 | CGK733 | 905973-89-9 | C23H18Cl3FN4O3S | 555.84 | No Development Reported |
| 45 | HY-114395 | NVS-ZP7-4 | 2349367-89-9 | C28H28FN5OS | 501.62 | No Development Reported |
| 46 | HY-108250 | (R)-Bicalutamide | 113299-40-4 | C18H14F4N2O4S | 430.37 | No Development Reported |
| 47 | HY-15568 | A-317491 | 475205-49-3 | C33H27NO8 | 565.57 | No Development Reported |
| 48 | HY-108689 | Broflanilide | 1207727-04-5 | C25H14BrF11N2O2 | 663.28 | No Development Reported |
| 49 | HY-124772 | BI-2545 | 2162961-71-7 | C23H19F6N5O3 | 527.42 | No Development Reported |
| 50 | HY-135230 | LY2444296 | 1346133-11-6 | C24H22F2N2O2 | 408.44 | No Development Reported |
| 51 | HY-114174 | Fmoc-Ala-Glu-Asn-Lys-NH2 | 220701-06-4 | C33H43N7O9 | 681.74 | No Development Reported |
| 52 | HY-114263 | NXT629 | 1454925-59-7 | C35H39N5O3S | 609.78 | No Development Reported |
| 53 | HY-12746 | DC-05 | 890643-16-0 | C25H25N3O | 383.49 | No Development Reported |
| 54 | HY-103628 | PROTAC CDK9 Degrader-1 | 2118356-96-8 | C33H35N5O7 | 613.66 | No Development Reported |
| 55 | HY-13928 | GW0742 | 317318-84-6 | C21H17F4NO3S2 | 471.49 | No Development Reported |
| 56 | HY-115521 | Jarin-1 | 1212704-51-2 | C28H29N3O4 | 471.55 | No Development Reported |
| 57 | HY-103410 | Carmoxirole (hydrochloride) | 115092-85-8 | C24H27ClN2O2 | 374.48 | No Development Reported |
| 58 | HY-100518 | T-26c | 869296-13-9 | C24H21N3O6S | 479.51 | No Development Reported |
| 59 | HY-12683 | BPTES | 314045-39-1 | C24H24N6O2S3 | 524.68 | No Development Reported |
| 60 | HY-108588 | NS5806 | 426834-69-7 | C16H8Br2F6N6O | 574.07 | No Development Reported |
| 61 | HY-129600 | MYCi361 | 2289690-31-7 | C26H16ClF9N2O2 | 594.86 | No Development Reported |
| 62 | HY-10171 | NPS-2143 (hydrochloride) | 324523-20-8 | C24H26Cl2N2O2 | 408.92 | No Development Reported |
| 63 | HY-117766 | PC945 | 1931946-73-4 | C38H37F3N6O3 | 682.73 | No Development Reported |
| 64 | HY-12439 | ML380 | 1627138-52-6 | C23H25F3N4O3S | 494.53 | No Development Reported |
| 65 | HY-15650 | SGC0946 | 1561178-17-3 | C28H40BrN7O4 | 618.57 | No Development Reported |
| 66 | HY-119377 | UPGL00004 | 1890169-95-5 | C25H26N8O2S2 | 534.66 | No Development Reported |
| 67 | HY-101120 | 666-15 | 1433286-70-4 | C33H31Cl2N3O5 | 584.06 | No Development Reported |
| 68 | HY-129681 | MCL-1/BCL-2-IN-1 |  | C31H27BrN2O3S | 587.53 | No Development Reported |
| 69 | HY-105917 | Endovion | 265646-85-3 | C16H9BrF6N6O | 495.18 | No Development Reported |
| 70 | HY-13491 | GNF-5837 | 1033769-28-6 | C28H21F4N5O2 | 535.49 | No Development Reported |
| 71 | HY-138407 | PD-1/PD-L1-IN 7 | 2374856-75-2 | C34H36Cl2N8O4 | 691.61 | No Development Reported |
| 72 | HY-120272 | SMAP-2 | 1809068-70-9 | C27H27F3N2O4S | 532.57 | No Development Reported |
| 73 | HY-100522 | FMK 9a | 1955550-51-2 | C23H21FN2O3 | 392.42 | No Development Reported |
| 74 | HY-111496 | Sulfo-NHS-SS-Biotin (sodium) | 325143-98-4 | C19H27N4NaO9S4 | 584.71 | No Development Reported |
| 75 | HY-110261 | GS143 | 916232-21-8 | C28H19FN2O4 | 466.46 | No Development Reported |
| 76 | HY-108625 | SHA 68 | 847553-89-3 | C26H24FN3O3 | 445.49 | No Development Reported |
| 77 | HY-101283 | HCH6-1 | 1435265-06-7 | C28H27N3O4 | 469.53 | No Development Reported |
| 78 | HY-117389 | Homocarbonyltopsentin | 172286-77-0 | C21H14N4O3 | 370.36 | No Development Reported |
| 79 | HY-134772 | AS1810722 | 909561-15-5 | C25H25F2N7O | 477.51 | No Development Reported |
| 80 | HY-135813 | LtaS-IN-1 | 877950-01-1 | C24H17N3O5 | 427.41 | No Development Reported |
| 81 | HY-135868 | Mito-apocynin (C2) | 1254044-41-1 | C28H27BrNO3P | 456.49 | No Development Reported |
| 82 | HY-112904 | XRK3F2 | 2375193-43-2 | C23H24ClF2NO3 | 399.43 | No Development Reported |
| 83 | HY-107723 | CGP71683 hydrochloride | 192322-50-2 | C26H30ClN5O2S | 475.61 | No Development Reported |
| 84 | HY-15274 | L-798106 | 244101-02-8 | C27H22BrNO4S | 536.44 | No Development Reported |
| 85 | HY-10871 | Otenabant | 686344-29-6 | C25H25Cl2N7O | 510.42 | No Development Reported |
| 86 | HY-14221A | Siramesine (hydrochloride) | 224177-60-0 | C30H32ClFN2O | 454.58 | No Development Reported |
| 87 | HY-112895 | UT-155 | 2031161-35-8 | C20H15F4N3O2 | 405.35 | No Development Reported |
| 88 | HY-12964 | SGI-7079 | 1239875-86-5 | C26H26FN7 | 455.53 | No Development Reported |
| 89 | HY-15623 | Hoechst 33258 analog | 258843-62-8 | C29H30N6O3 | 510.59 | No Development Reported |
| 90 | HY-13108 | Bz 423 | 216691-95-1 | C27H21ClN2O2 | 440.92 | No Development Reported |
| 91 | HY-107536 | ML 145 | 1164500-72-4 | C24H22N2O5S2 | 482.57 | No Development Reported |
| 92 | HY-12835 | S1P1 Agonist III | 1324003-64-6 | C21H16F3N3O3 | 415.37 | No Development Reported |
| 93 | HY-112701 | CCR6 inhibitor 1 | 2437547-04-9 | C24H23F3N4O3S | 504.52 | No Development Reported |
| 94 | HY-120210 | XY018 | 1873358-87-2 | C23H15F7N2O4 | 516.37 | No Development Reported |
| 95 | HY-116564 | Lotilaner | 1369852-71-0 | C20H14Cl3F6N3O3S | 596.76 | No Development Reported |
| 96 | HY-103695 | CD73-IN-1 | 2132396-40-6 | C18H17N3O4S | 371.41 | No Development Reported |
| 97 | HY-100892 | MX69 | 1005264-47-0 | C27H26N2O4S | 474.57 | No Development Reported |
| 98 | HY-112895A | (R)-UT-155 | 2031161-54-1 | C20H15F4N3O2 | 405.35 | No Development Reported |
| 99 | HY-136555 | GSK717 | 1595278-21-9 | C28H28N4O2 | 452.55 | No Development Reported |
| 100 | HY-108468 | KL001 | 309928-48-1 | C21H22N2O4S | 398.48 | No Development Reported |
| 101 | HY-133016A | (R)-M8891 | 2575547-29-2 | C20H17F2N3O3 | 385.36 | No Development Reported |
| 102 | HY-108434 | Ceapin-A7 | 2323027-38-7 | C20H12F6N4O3 | 470.32 | No Development Reported |
| 103 | HY-120635 | BMS-1001 (hydrochloride) | 2113650-04-5 | C35H35ClN2O7 | 594.65 | No Development Reported |
| 104 | HY-131003 | Taletrectinib | 1505515-69-4 | C29H34FN5O5 | 405.47 | No Development Reported |
| 105 | HY-10655 | Palosuran | 540769-28-6 | C25H30N4O2 | 418.53 | No Development Reported |
| 106 | HY-112136 | TAO Kinase inhibitor 1 | 850467-66-2 | C25H24N2O2 | 384.47 | No Development Reported |
| 107 | HY-122913 | Borussertib | 1800070-77-2 | C36H32N6O3 | 596.68 | No Development Reported |
| 108 | HY-124711 | TBOPP | 1996629-79-8 | C24H21F3N2O4S | 490.49 | No Development Reported |
| 109 | HY-124944 | APS6-45 | 2188236-41-9 | C23H16F8N4O3 | 548.39 | No Development Reported |
| 110 | HY-102060 | WM-8014 | 2055397-18-5 | C20H17FN2O3S | 384.42 | No Development Reported |
| 111 | HY-15556 | GSK269962A | 850664-21-0 | C29H30N8O5 | 570.60 | No Development Reported |
| 112 | HY-100609 | 4-P-PDOT | 134865-74-0 | C19H21NO | 279.38 | No Development Reported |
| 113 | HY-130606 | Nampt-IN-5 | 2380013-17-0 | C25H25N5O2 | 427.50 | No Development Reported |
| 114 | HY-13200 | BRL-15572 (dihydrochloride) | 193611-72-2 | C25H29Cl3N2O | 406.95 | No Development Reported |
| 115 | HY-15193B | EMD638683 (S-Form) | 1184940-46-2 | C18H18F2N2O4 | 364.34 | No Development Reported |
| 116 | HY-112679 | GLP-1 receptor agonist 2 | 2230197-64-3 | C30H31ClFN5O4 | 580.05 | No Development Reported |
| 117 | HY-129578 | Asperphenamate | 63631-36-7 | C32H30N2O4 | 506.59 | No Development Reported |
| 118 | HY-100131 | GSK481 | 1622849-58-4 | C21H19N3O4 | 377.39 | No Development Reported |
| 119 | HY-102039 | CDK9-IN-8 | 2105956-51-0 | C31H32FN7O3 | 569.63 | No Development Reported |
| 120 | HY-120793 | CMC2.24 | 1255639-43-0 | C26H21NO5 | 427.45 | No Development Reported |
| 121 | HY-111108 | LDH-IN-1 | 1964515-43-2 | C30H26N4O4S2 | 570.68 | No Development Reported |
| 122 | HY-12382 | NMS-P715 | 1202055-32-0 | C35H39F3N8O3 | 676.73 | No Development Reported |
| 123 | HY-15193 | EMD638683 | 1181770-72-8 | C18H18F2N2O4 | 364.34 | No Development Reported |
| 124 | HY-112094 | WNK-IN-11 | 2123489-30-3 | C21H21Cl2N5OS | 462.40 | No Development Reported |
| 125 | HY-120327 | KY-226 | 1621673-53-7 | C27H31NO3S2 | 481.67 | No Development Reported |
| 126 | HY-136657 | SC-43 | 1400989-25-4 | C21H13ClF3N3O2 | 431.80 | No Development Reported |
| 127 | HY-136431 | BC-DXI-843 | 2421117-98-6 | C28H26N4O4S2 | 546.66 | No Development Reported |
| 128 | HY-14973 | Vidupiprant | 1169483-24-2 | C28H27Cl2FN2O6S | 609.49 | No Development Reported |
| 129 | HY-137655A | BMS-P5 (free base) | 1550371-22-6 | C27H32N6O2 | 472.58 | No Development Reported |
| 130 | HY-11048 | NS11394 | 951650-22-9 | C23H19N3O | 353.42 | No Development Reported |
| 131 | HY-103320A | Calhex 231 (hydrochloride) | 2387505-78-2 | C25H28Cl2N2O | 406.95 | No Development Reported |
| 132 | HY-11077 | SR 146131 | 221671-61-0 | C32H36ClN3O5S | 610.16 | No Development Reported |
| 133 | HY-13103 | NS 11021 | 956014-19-0 | C16H9BrF6N6S | 511.24 | No Development Reported |
| 134 | HY-100017 | BAY-876 | 1799753-84-6 | C24H16F4N6O2 | 496.42 | No Development Reported |
| 135 | HY-138627A | AST5902 (trimesylate) |  | C30H41F3N8O11S3 | 554.57 | No Development Reported |
| 136 | HY-15701 | Leukadherin-1 | 344897-95-6 | C22H15NO4S2 | 421.49 | No Development Reported |
| 137 | HY-12846 | CCT196969 | 1163719-56-9 | C27H24FN7O3 | 513.52 | No Development Reported |
| 138 | HY-15701B | ADH-503 | 2055362-74-6 | C27H28N2O5S2 | 421.49 | No Development Reported |
| 139 | HY-101787 | FTBMT | 1358575-02-6 | C19H16F4N4O | 392.35 | No Development Reported |
| 140 | HY-136571 | GSK046 | 2474876-09-8 | C23H27FN2O4 | 414.47 | No Development Reported |
| 141 | HY-107633 | A 1120 | 1152782-19-8 | C20H19F3N2O3 | 392.37 | No Development Reported |
| 142 | HY-14562 | TBPB | 634616-95-8 | C25H32N4O | 404.55 | No Development Reported |
| 143 | HY-12776 | GSK805 | 1426802-50-7 | C23H18Cl2F3NO4S | 532.36 | No Development Reported |
| 144 | HY-12815A | MCC950 (sodium) | 256373-96-3 | C20H23N2NaO5S | 404.48 | No Development Reported |
| 145 | HY-135674 | SR-318 | 2413286-32-3 | C27H33N5O2 | 459.58 | No Development Reported |
| 146 | HY-103665 | STING agonist-3 | 2138299-29-1 | C37H42N12O6 | 750.81 | No Development Reported |
| 147 | HY-100519 | NVS-PAK1-1 | 1783816-74-9 | C23H25ClF3N5O | 479.93 | No Development Reported |
| 148 | HY-100733 | 4E2RCat | 432499-63-3 | C22H14ClNO4S2 | 455.93 | No Development Reported |
| 149 | HY-114208A | BI-9321 (trihydrochloride) | 2387510-87-2 | C22H24Cl3FN4 | 360.43 | No Development Reported |
| 150 | HY-124719 | hSMG-1 inhibitor 11j | 1402452-15-6 | C27H28ClN7O3S | 566.07 | No Development Reported |
