# Supplementary material for: A novel penicillin-binding protein inhibitor with unprecedented intracellular activity eradicates multiple pathogenic bacteria
Source: PLoS Pathog. 2026 Jul 16;22(7):e1014242. doi: 10.1371/journal.ppat.1014242 (PMC13374901; doi:10.1371/journal.ppat.1014242)
Supplement: S2 Table — Mouse Blood Pressure Measurements (mmHg). (DOCX) [file ppat.1014242.s003.docx]

**A novel penicillin-binding protein inhibitor with unprecedented intracellular activity eradicates multiple pathogenic bacteria**

**S2 Table. Mouse Blood Pressure Measurements (mmHg).**

| Parameter (mmHg) | Control Group (PBS) | RS 17053 Group  (10 mg/kg/day) | 1. value   (Unpaired t-test) |
| --- | --- | --- | --- |
| Systolic BP | 104.00 ± 3.9 | 99.53 ± 6.1 | 0.38 |
| Mean Arterial Pressure | 79.00 ± 3.5 | 76.20 ± 5.2 | 0.64 |
| Diastolic BP | 65.93 ± 3.4 | 64.47 ± 5.1 | 0.80 |
