# Supplementary material for: A novel penicillin-binding protein inhibitor with unprecedented intracellular activity eradicates multiple pathogenic bacteria
Source: PLoS Pathog. 2026 Jul 16;22(7):e1014242. doi: 10.1371/journal.ppat.1014242 (PMC13374901; doi:10.1371/journal.ppat.1014242)
Supplement: S3 Table — Broad-spectrum antimicrobial activity of RS 17053. (DOCX) [file ppat.1014242.s004.docx]

**A novel penicillin-binding protein inhibitor with unprecedented intracellular activity eradicates multiple pathogenic bacteria**

**S3 Table. Broad-spectrum antimicrobial activity of RS 17053.**

| Strain information | MID (mm) | MIC (μg/mL) | MBC (μg/mL) |
| --- | --- | --- | --- |
| *B.melitensis* TZ | 42.41 | 0.25-0.5 | 2 |
| *B.suis* S2 | 22.36 | 2-4 | 8 |
| *B.abortos* A19 | 24.63 | 2-4 | 8 |
| *B.melitensis* M5-90 | 24.68 | 1-2 | 4 |
| *S. typhimurium* SL1344 | 23.12 | 2-4 | 8 |
| *S. aureus* ATCC29213 | 19.08 | 1-2 | 4 |
| MRSA | 18.01 | 2-4 | 8 |
| *E. coli* ATCC 25922 | 23.42 | 2-4 | 8 |
| HPS ATCC 70025 | 25.23 | 2 | 8 |
| RA-YM | 30.96 | 0.25-0.5 | 2 |

Tablenotes: Quality control is the MIC value of gentamicin against *E. coli* ATCC 25922, which all meet the requirements of CLSI. All experiments were guaranteed 3 biological replicates.
