# Supplementary material for: A novel penicillin-binding protein inhibitor with unprecedented intracellular activity eradicates multiple pathogenic bacteria
Source: PLoS Pathog. 2026 Jul 16;22(7):e1014242. doi: 10.1371/journal.ppat.1014242 (PMC13374901; doi:10.1371/journal.ppat.1014242)
Supplement: S4 Table — RT-qPCR assays primers. (DOCX) [file ppat.1014242.s005.docx]

**A novel penicillin-binding protein inhibitor with unprecedented intracellular activity eradicates multiple pathogenic bacteria**

**S4 Table. RT-qPCR assays primers.**

| Gene name | Primer sequence5’-3’ | Fragment length (bp) |
| --- | --- | --- |
| TZ-MrcA-F | aaggatgaaccggcctgc | 189 |
| TZ-MrcA-R | ccagaaagccgccgaaga |  |
| TZ-OppF-F | ggttgaaaaggcgctgagc | 191 |
| TZ-OppF-R | cagattgcgcttccgctct |  |
| TZ-Omp19-R | cgtcgcgatcttgcagct | 190 |
| TZ-Omp19-R | aggcacggtgcagaaagg |  |
| MRSA-MecA-F | agcacttgtaagcacaccttca | 143 |
| MRSA-MecA-R | tttgagttgaacctggtgaagttgt |  |
| MRSA-BlaZ-F | cgaaagcagcaggcgttg | 157 |
| MRSA-BlaZ-R | aacaacaaaattataaacgaaatcggtgg |  |
| MRSA-EF-Tu-F | tgtgttcacgagtttgtggca | 220 |
| MRSA-EF-Tu-R | tacgacatgattgacaacgctcc |  |
